# Effects of Several Classes of Voltage-Gated Ion Channel Conductances on Gamma and Theta Oscillations in a Hippocampal Microcircuit Model

**DOI:** 10.3389/fncom.2021.630271

**Published:** 2021-04-01

**Authors:** Chris Olteanu, Forough Habibollahi, Chris French

**Affiliations:** ^1^Melbourne Brain Centre, The University of Melbourne, Parkville, VIC, Australia; ^2^Department of Biomedical Engineering, The University of Melbourne, Parkville, VIC, Australia; ^3^Department of Medicine, The Royal Melbourne Hospital, The University of Melbourne, Parkville, VIC, Australia

**Keywords:** neural oscillations, theta rhythmogenesis, gamma rhythmogenesis, voltage-gated ion channels, pyramidal-interneuron-gamma network, hippocampal neural network

## Abstract

Gamma and theta oscillations have been functionally associated with cognitive processes, such as learning and memory. Synaptic conductances play an important role in the generation of intrinsic network rhythmicity, but few studies have examined the effects of voltage-gated ion channels (VGICs) on these rhythms. In this report, we have used a pyramidal-interneuron-gamma (PING) network consisting of excitatory pyramidal cells and two types of inhibitory interneurons. We have constructed a conductance-based neural network incorporating a persistent sodium current (*I*_*NaP*_), a delayed rectifier potassium current (*I*_*KDR*_), a inactivating potassium current (*I*_*A*_) and a hyperpolarization-activated current (*I*_*H*_). We have investigated the effects of several conductances on network theta and gamma frequency oscillations. Variation of all conductances of interest changed network rhythmicity. Theta power was altered by all conductances tested. Gamma rhythmogenesis was dependent on *I*_*A*_ and *I*_*H*_. The *I*_*KDR*_ currents in excitatory pyramidal cells as well as both types of inhibitory interneurons were essential for theta rhythmogenesis and altered gamma rhythm properties. Increasing *I*_*NaP*_ suppressed both gamma and theta rhythms. Addition of noise did not alter these patterns. Our findings suggest that VGICs strongly affect brain network rhythms. Further investigations *in vivo* will be of great interest, including potential effects on neural function and cognition.

## 1. Introduction

Synchronous oscillations in the brain, including hippocampal networks, have attracted much attention in recent years. These oscillations can be investigated *in vivo* and *in vitro*, and more recently can be modeled *in silico* due to the wide availability of advanced desktop processors and simulation software. These rhythmic activities are believed to represent temporal links between compartments of larger neural ensembles (Colgin and Moser, 2010). Increasing evidence suggests that brain oscillations have a key function in mechanisms of sensory-cognitive processes and that some forms of cognitive neuropathology may represent “rhythmopathies,” i.e., disorders of normal rhythmicity (Mably and Colgin, 2018). Abnormal neural oscillations have been reported in schizophrenia (Uhlhaas and Singer, 2015), cognitive and motor phenotypes of Parkinson's disease (Oswal et al., 2013), as well as mild cognitive impairment and Alzheimer's disease (Başar, 2013; Başar-Eroğlu et al., 2013; Vecchio et al., 2013; Yener and Başar, 2013). Thus, understanding the basis of these oscillations may improve the functional deficits of these diseases as well as potential therapeutic targets.

The hippocampus plays a significant role in learning and memory encoding and retrieval (Nadel and Moscovitch, 1997; Douchamps et al., 2013). These functions are related to hippocampal oscillatory activities (Xu et al., 2004). Hippocampal gamma (40–80 Hz) and theta (3–8 Hz) rhythms have been specifically implicated in cognitive functions (Rutishauser et al., 2010; Wang, 2010; Lisman and Jensen, 2013). Theta rhythm is also believed to be significant in exploratory movement in rats (Buzsáki, 2002). Synaptic currents and network connectivity have significant roles in network rhythms (Wang, 2010). In addition, voltage-gated conductances play an important role in network rhythmogenesis which has been less extensively explored. Voltage-gated conductances, such as the hyperpolarization activated non-specific cation current (*I*_*H*_) and the persistent sodium current (*I*_*NaP*_), have been shown to affect theta (Xu et al., 2004) and gamma rhythms (Wang, 1993), respectively. This implies a potent role of *I*_*H*_ and *I*_*NaP*_ in rhythmogenesis and potential modulatory targets. To further explore the effects of voltage-gated ion channels (VGICs) on rhythmicity, we constructed a pyramidal-interneuron-gamma (PING) network model containing several voltage-gated conductances and three distinct populations of neurons: excitatory pyramidal cells (E-cells), oriens lacunosum moleculare interneurons (O-cells), and inhibitory interneurons (I-cells). In this form of gamma, individual E-cells fire at or near gamma frequency, and their active participation is crucial; that is, the E-cells drive and synchronize the I-cells, and the I-cells gate and synchronize the E-cells (Traub et al., 1997; Börgers et al., 2005). In this network, there is a very strong connectivity between excitatory and inhibitory sub-populations, with such short rise times that an increase in E-cell spiking quickly evokes a surge in spiking of the I-cells (Rich et al., 2017). The resulting pulse of inhibitory input to the E-cells leads them toward synchrony. When inhibition wears off, the E-cells resume spiking, causing the cycle to repeat. Such strong connectivities can then create a 1:1 bursting ratio between inhibitory and excitatory cells that is a hallmark of classic PING theory (Kopell et al., 2010). A similar ratio in excitatory and inhibitory firing rates is supported by *in vitro* experimental evidence (Whittington et al., 2000). In such a model, the external drives play a critical role in generating the PING activity as well (Börgers and Kopell, 2005). A very weak drive current to the E-cells, similar to a very strong drive to I-cells, can abolish the PING rhythms. In these scenarios, I-cells can synchronize at frequencies too high to be entrained by the E-cells, or act asynchronously, which inhibits E-cell activity and hence, the synchronous rhythms vanish. Nevertheless, there is a setting in this parameter space where both suppression of the E-cells by asynchronous activity of the I-cells and PING may occur. In a setting where E-cells and I-cells produce oscillations of roughly the same frequency, in the absence of O-cells, the system is pushed toward instability in the presence of noise (see Börgers and Kopell, 2005). The O-cell spikes affect both E-cell and I-cell populations. O-cells are a type of inhibitory neurons in the hippocampus that gate information flow, while firing phase-locked to and taking part in generating theta rhythms (Sekulić and Skinner, 2017). Their firing activity during the trough of theta rhythms is recorded in the local field potentials in the pyramidal layer of the CA1 region of the hippocampus (Klausberger et al., 2003; Varga et al., 2012). These cells target distal dendrites of pyramidal cells in CA1 and are known to express hyperpolarization-activated inward channels (Maccaferri and McBain, 1996) that enables them to express post-inhibitory rebound spiking and contribute to *in vivo* theta rhythms (Sekulić and Skinner, 2017).

By utilizing this model in the appropriate parameter regime, we report effects on theta and gamma oscillations in response to changes in a range of VGIC conductance amplitudes. Finally, we examine whether the nature and degree of alterations in VGIC conductances affect rhythmogenesis of theta and gamma oscillations.

## 2. Methods

### 2.1. Noiseless Simulations

**Figure 1:**
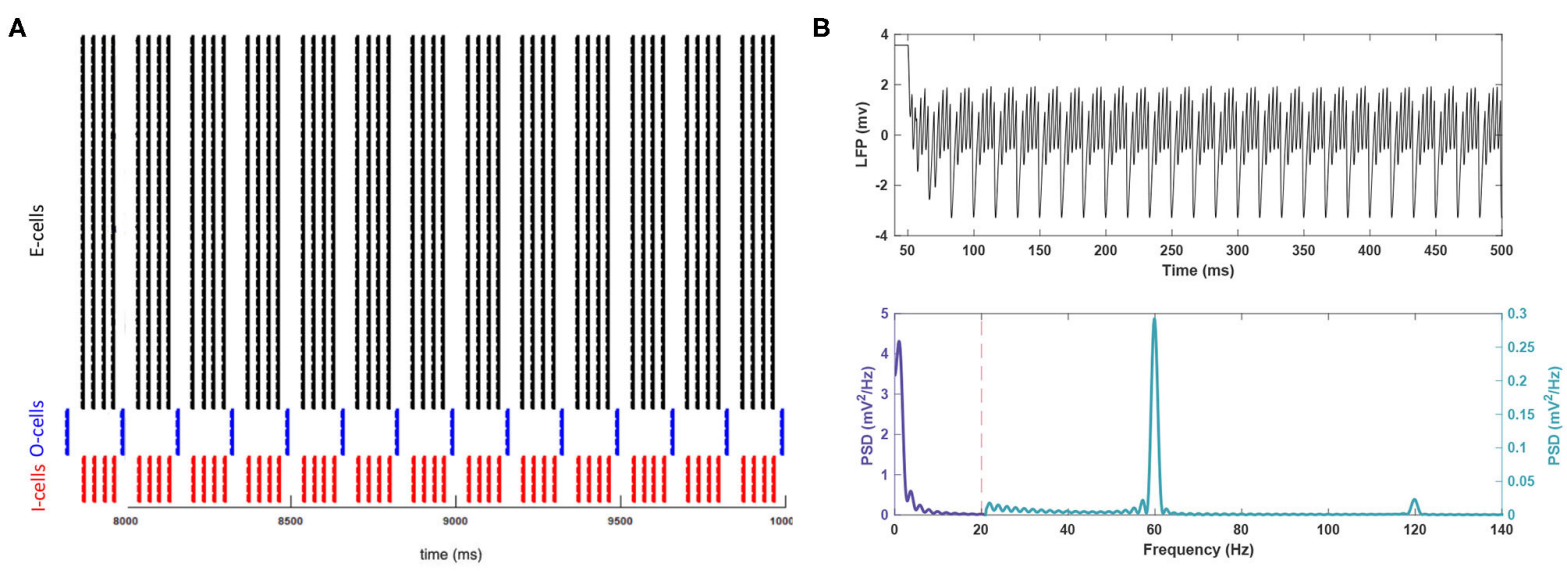
The activity of the network in baseline conditions. **(A)** The raster plot indicates the spike activity of individual neurons from different populations in the last 2 s of the simulation. **(B)** (Top) For better readability, the LFP signal is shown only for the duration of 50–500 ms of the simulation after the system reaches steady-state. This period is long enough to reach the steady state. (Bottom) The theta rhythm and the gamma nested within it are noticeable in the power spectrum for the signal. Note the different scales used for each frequency band.

A model developed by Kopell et al. (2010) (see also Gloveli et al., 2005; Tort et al., 2007) was implemented in *NEURON*. This model has been used in recent studies (Scheffer-Teixeira and Tort, 2016; Keeley et al., 2017; Jansen et al., 2021), and in this work was augmented with a persistent sodium conductance (*g*_*NaP*_) added to the excitatory pyramidal cells (E-cells). We then constructed a network consisting of 40 E-cells, five oriens lacunosum moleculare interneurons (O-cells), and five inhibitory interneurons (I-cells) within CA1 hippocampus. A persistent sodium conductance (*g*_*NaP*_) was incorporated in the E-cells, and was utilized along with a delayed rectifier potassium conductance in each cell type (*g*_*KDRe*_ for E-cells, *g*_*KDRi*_ for I-cells, and *g*_*KDRo*_ for O-cells), a inactivating potassium conductance (*g*_*A*_) and a hyperpolarization-activated current conductance (*g*_*H*_) in the O-cells. Initial simulations were performed without added noise, with baseline values of *g*_*A*_= 16, *g*_*Na*_ = 100, *g*_*H*_ = 12, *g*_*KDRe*_ = 80, *g*_*KDRi*_ = 9, and *g*_*KDRo*_ = 23 ($\frac{mS}{cm^{2}}$). The *g*_*NaP*_ was set to 2*e*^−5^ $\frac{mS}{cm^{2}}$ (Hsu et al., 2018). The network was driven by currents applied somatically to the E, I, and O-cell populations (Kopell et al., 2010) with values of *I*_*E*_ = 0.8, *I*_*I*_ = 0.8, and *I*_*O*_ = 3 in $\frac{\mu A}{cm^{2}}$. The details of the implemented model and the parameter settings can be found in the Supplementary Material of this article. Local field potentials (LFPs) were measured by using an E cell with no drive currents as a probe (see Kopell et al., 2010). Power spectra calculations on LFPs were performed by high and low pass filtering at 0.5 and 100 Hz, respectively. The *spectrum.periodogram* function from the Signal Processing Toolbox in MATLAB (Chambers et al., 2012) was utilized to measure the power spectrum. The activity of the network with the baseline settings is shown in Figure 1 and serves as control data.

Simulations of 10,000 ms were performed on an *Acer Nitro 5* computer with a quadcore Intel Core *i5-8300H CPU* and *8GB RAM*, running Windows 10. In each run, one conductance value was modified and all remaining conductance values were kept constant to observe shifts in spectra after the model reached steady-state once again. For *g*_*A*_, *g*_*H*_, *g*_*KDRe*_, *g*_*KDRi*_, and *g*_*KDRo*_ trials, conductances were set to ratios of 0, 0.05, 0.5, 0.8, 1.5, 1.8, and 2 g, whereby *g* represents baseline conductance. In *g*_*NaP*_ trials, larger incremental steps of conductance values were required to observe network changes. Thus, the set of 0, 0.05, 20, 35, 50, 65, and 80 g was employed for *g*_*NaP*_ trials.

### 2.2. Noisy Simulations

Additional simulations were performed with noise incorporated. The applied current was set to *I*_*E*_ = 0.8 +*W*, whereby *W* depicts a white noise process with an *SEM* (standard error of the mean) of 1.35*e*^−3^ $\frac{\mu A}{cm^{2}}$, so the external drive has deterministic and stochastic components. In order to determine the effects of conductance change on power spectra, 11 simulations were performed at each conductance level. For each set, runs were averaged to report peak power and frequency. Confidence intervals were generated for both mean peak power and frequency. For statistical analysis we conducted a two-tailed *Student's t-test* with 10 degrees of freedom and a significance level of *p* < 0.05 (i.e., 95% confidence levels). The results are reported as *mean* ± *standard error of the mean (SEM)*.

## 3. Results

### 3.1. Baseline Network Rhythmicity

Control simulations with no added noise are shown in Figure 1. The resulting network rhythmicity is consistent with previous studies of this PING network (Kopell et al., 2010), with clearly visible theta and gamma activity peaking at 3.70 and 59.89 Hz, respectively. Theta band power is more prominent than the gamma band (0.575 vs. 0.288 $\frac{mV^{2}}{Hz}$), which is also within expectations for gamma nested in theta *in vivo* (Colgin and Moser, 2010; Jacobson et al., 2013; Butler et al., 2016). There is a lesser peak present in higher frequency activity (e.g., fast gamma) around 120 Hz. However, this peak is not representing a true oscillation but a harmonic of the fundamental gamma peak at 60 Hz.

**Figure 2:**
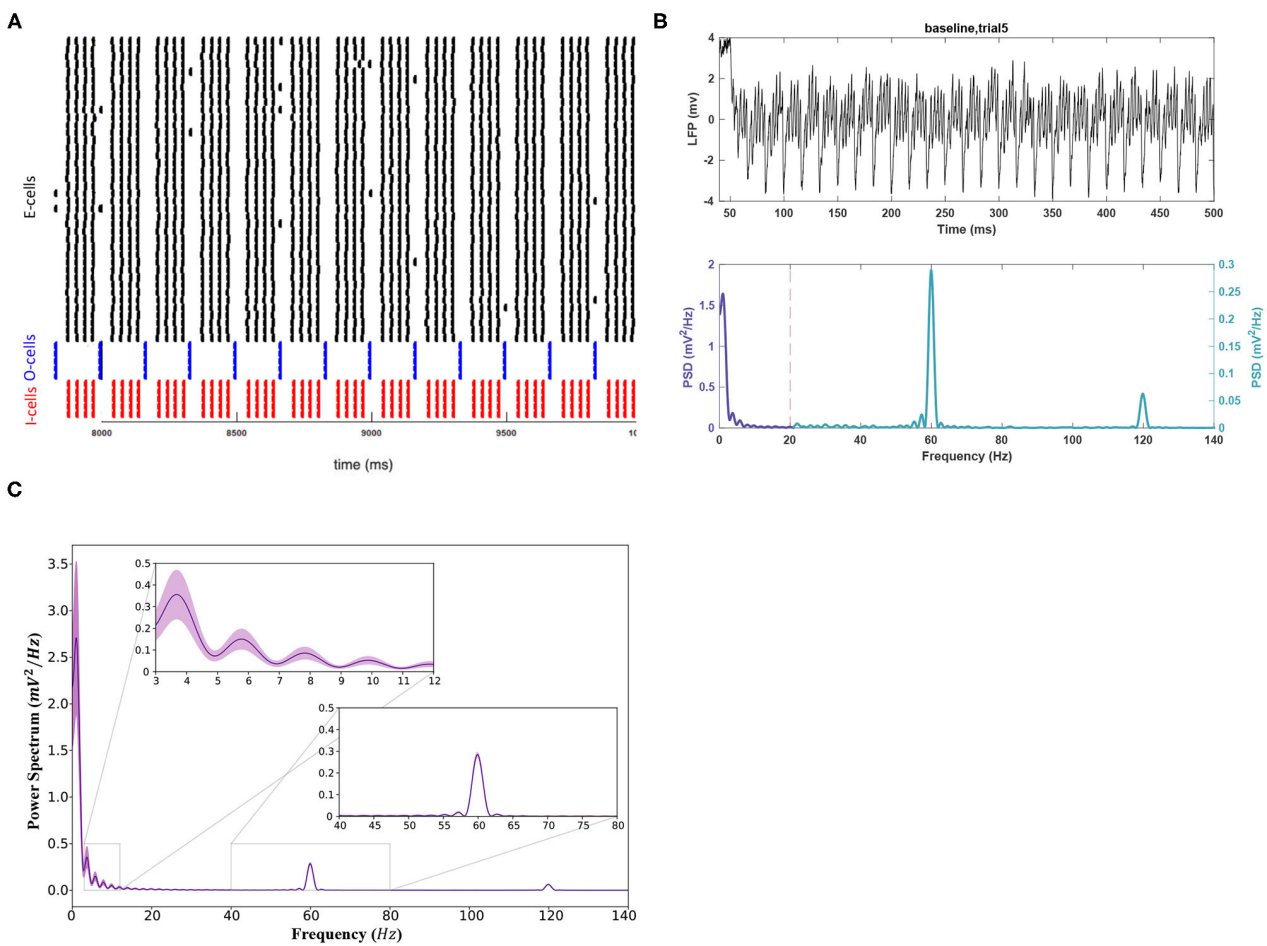
The activity of the network in baseline conditions after applying the white noise to the E-cell population. **(A)** The raster plot indicating the spike activity of individual neurons. **(B)** (Top) For better readability, the LFP signal is shown only for the duration of 50–500 ms of the simulation for a sample trial run. (Bottom) The theta rhythm and the gamma nested within it are noticeable in the power spectrum for the signal. Note the different scales used for each frequency band. **(C)** The mean power spectrum using 11 trial runs and the corresponding 95% confidence intervals around it using a *Student's t-test*.

Baseline simulations with noise are displayed in Figure 2. Figures 2A,B depict the baseline network activity. Figure 2C shows the mean power spectrum for all 11 trials. Only significant findings from the noisy experiments are reported. highlighting differences between noisy and noiseless trials. Theta power peaked with a value of 0.356 ± 0.111 $\frac{mV^{2}}{Hz}$ (Figure 2B), lower than the noiseless peak (0.5752). The gamma peak showed no significant shift in power and remained lower than theta (0.285 ± 0.010 $\frac{mV^{2}}{Hz}$, *p* < 0.05). Mean gamma and theta peak frequencies were also consistent with noise-free values, at 59.910 ± 0.033 and 3.766 ± 0.198 Hz, respectively. Figure 2C indicates that the *SEM* was greater in theta compared to gamma. Thus, the theta band appeared more sensitive to changes in noisy conditions.

### 3.2. Network Rhythmicity in Response to Variation of VGIC Conductance Amplitudes

**Figure 3:**
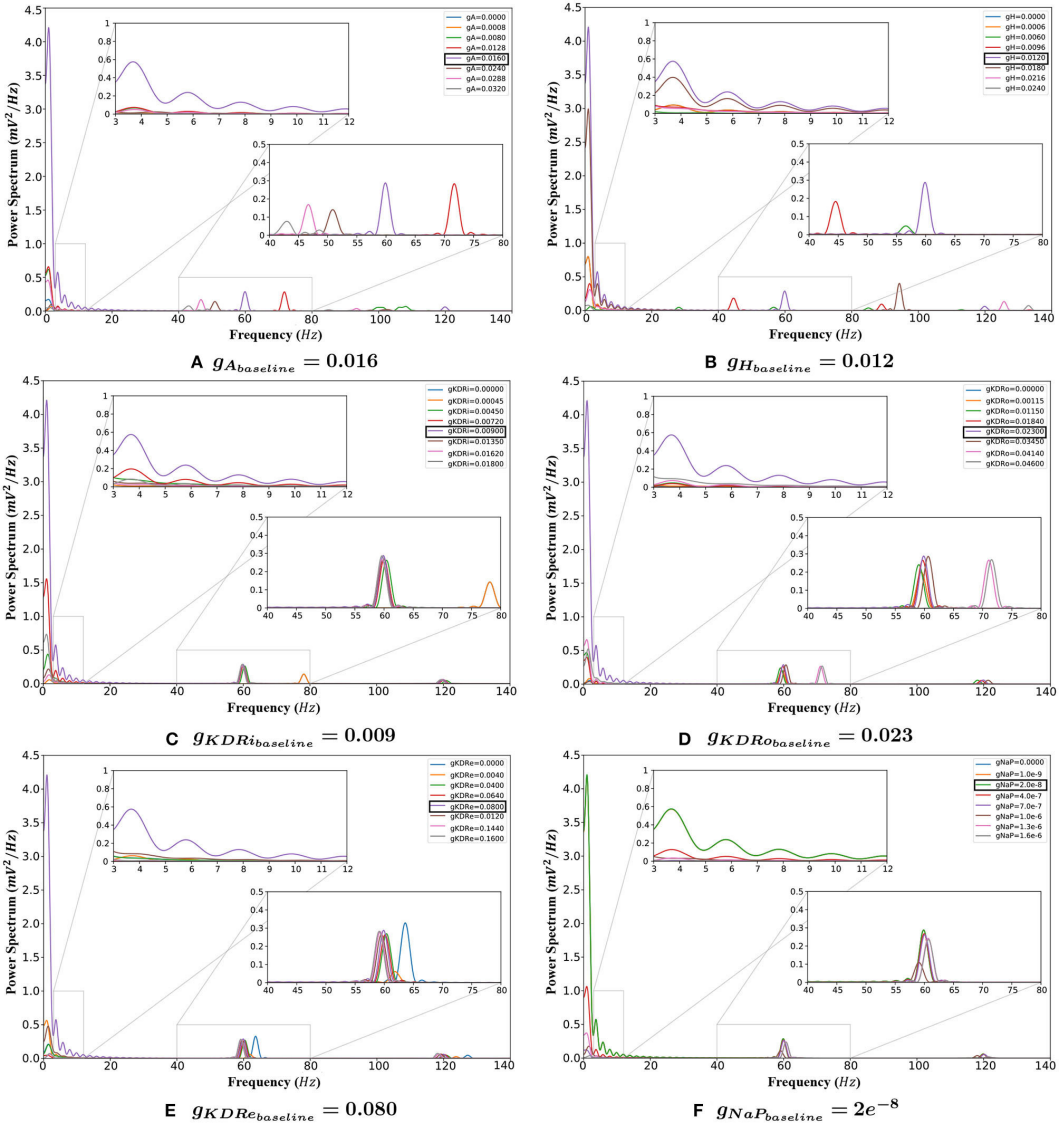
The behavior of the system with changing VGIC conductances, with closeups of the theta (3–12 Hz) and gamma (40–80 Hz) band frequency ranges. **(A)** Any change in the *g*_*A*_ value compared to baseline greatly reduces the power of oscillations in the theta range. It also causes the gamma rhythms to lower and vanish eventually. **(B)** Any variation from the baseline setting in the *g*_*H*_ reduces the power of both theta and gamma rhythms. **(C,D)** Deviations from baseline in the *g*_*KDR*_ value in both groups of inhibitory interneurons reduces the theta band power. Gamma rhythms also decrease but less significantly. **(E)** Conductance shifts in the potassium delayed rectifier in the E-cells weaken the theta band significantly. This current also weakens the gamma rhythm. **(F)** Lowering the value of *g*_*NaP*_ does not have any appreciable effect on the network rhythmicity. Increasing conductances, however, greatly lower the theta band power.

**Figure 4:**
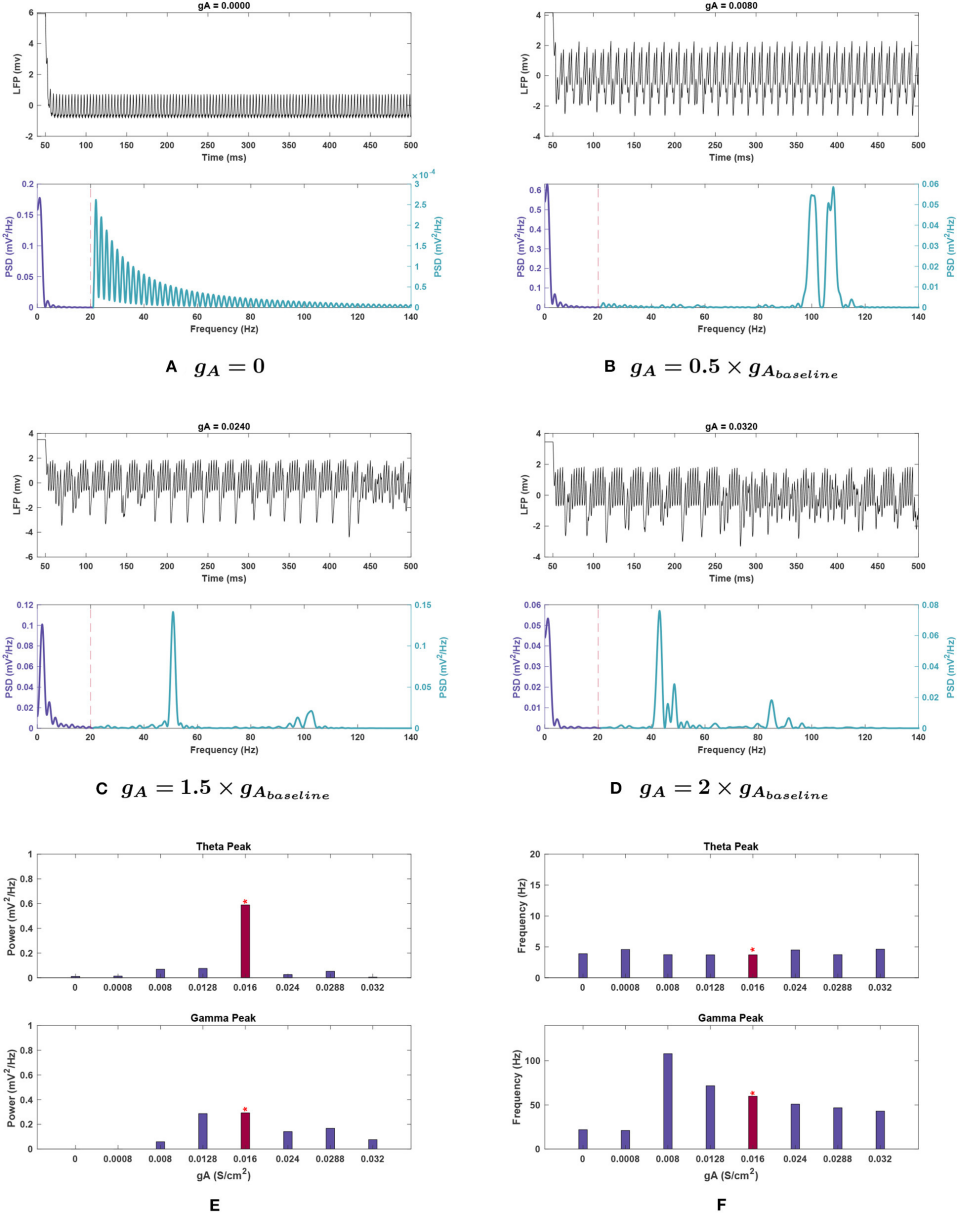
Impact of *g*_*A*_ on network rhythmicity. **(A–D)** Plots showing the effects of selected *g*_*A*_ values on the overall LFP signal (top) and power spectrum (bottom) of the network. **(E,F)** Graphical representation of how conductance values affect the **(E)** power and **(F)** frequency of peak rhythms in the theta (top) and gamma (bottom) bands. The red bar with the asterisk sign above indicates the results for the baseline value of the conductance under investigation. The results demonstrate that the *A* current has a very significant effect on both theta and gamma rhythm generation. It may be worth noting that decreasing this conductance seems to push the gamma rhythm toward higher frequencies before dropping off. Theta rhythms are weakened in all cases compared to the baseline in Figure 1.

**Figure 5:**
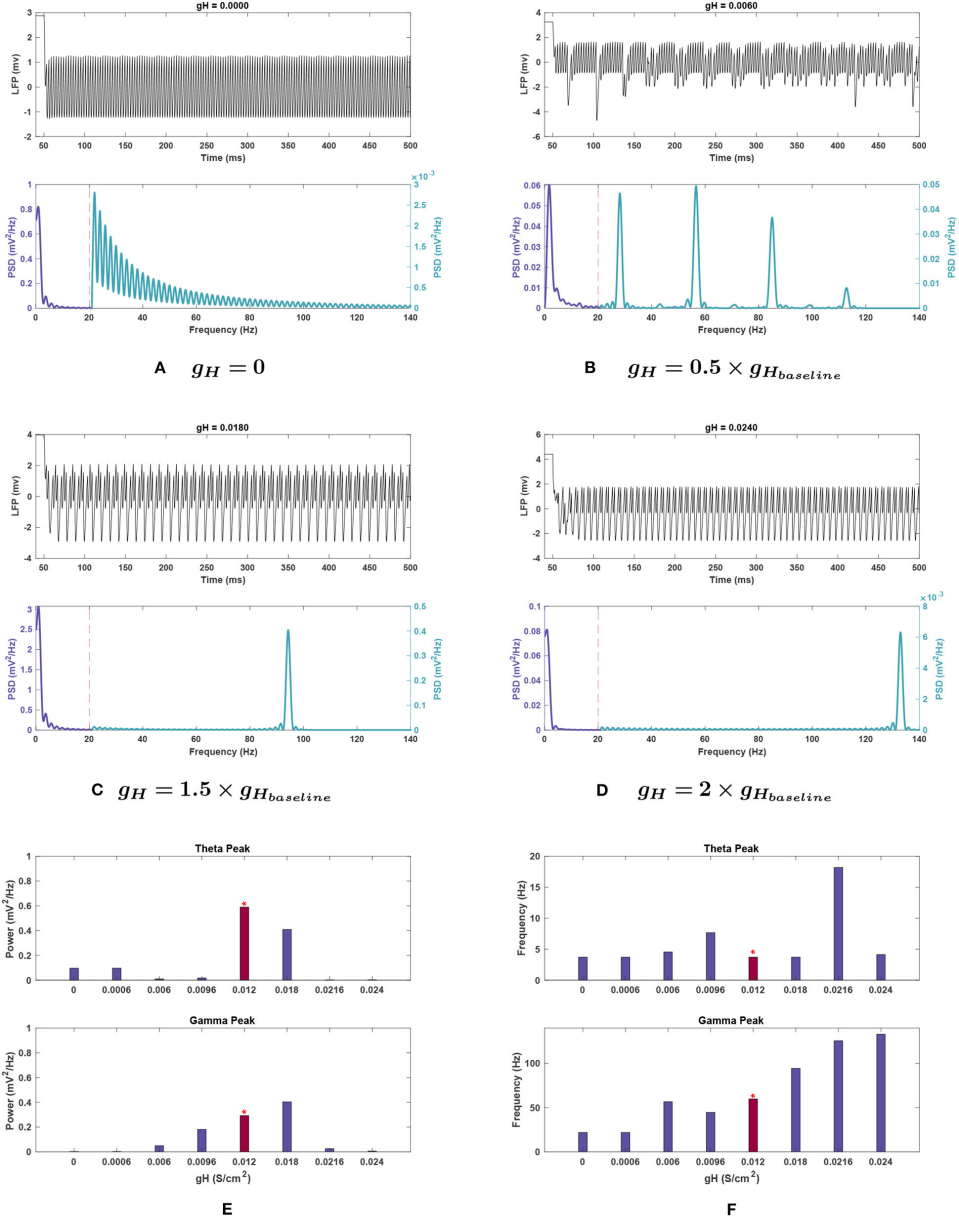
Impact of *g*_*H*_ on network rhythmicity. **(A–D)** Network LFP signal (top) and power spectra (bottom) at selected *g*_*H*_ values. **(E,F)** Changes in **(E)** power and **(F)** frequency in response to *g*_*H*_ for both theta (top) and gamma (bottom) peaks. The red bar with the asterisk sign indicates the results for the baseline value of *g*_*H*_. The results illustrate that the presence of *H* current is necessary for the generation of the gamma oscillations, while theta rhythms conversely seem to be lost at high conductance. Increased *g*_*H*_ values will initially shift the gamma oscillations more toward fast gamma. Although further increases appear to shift the gamma peak toward higher values, it is vital to note that the gamma power is vanished at these values; Hence, the detected peaks are not of any significance. Lowering *g*_*H*_ values also decreases the gamma power. Theta peaks are all greatly reduced compared to the power seen in Figure 1.

**Figure 6:**
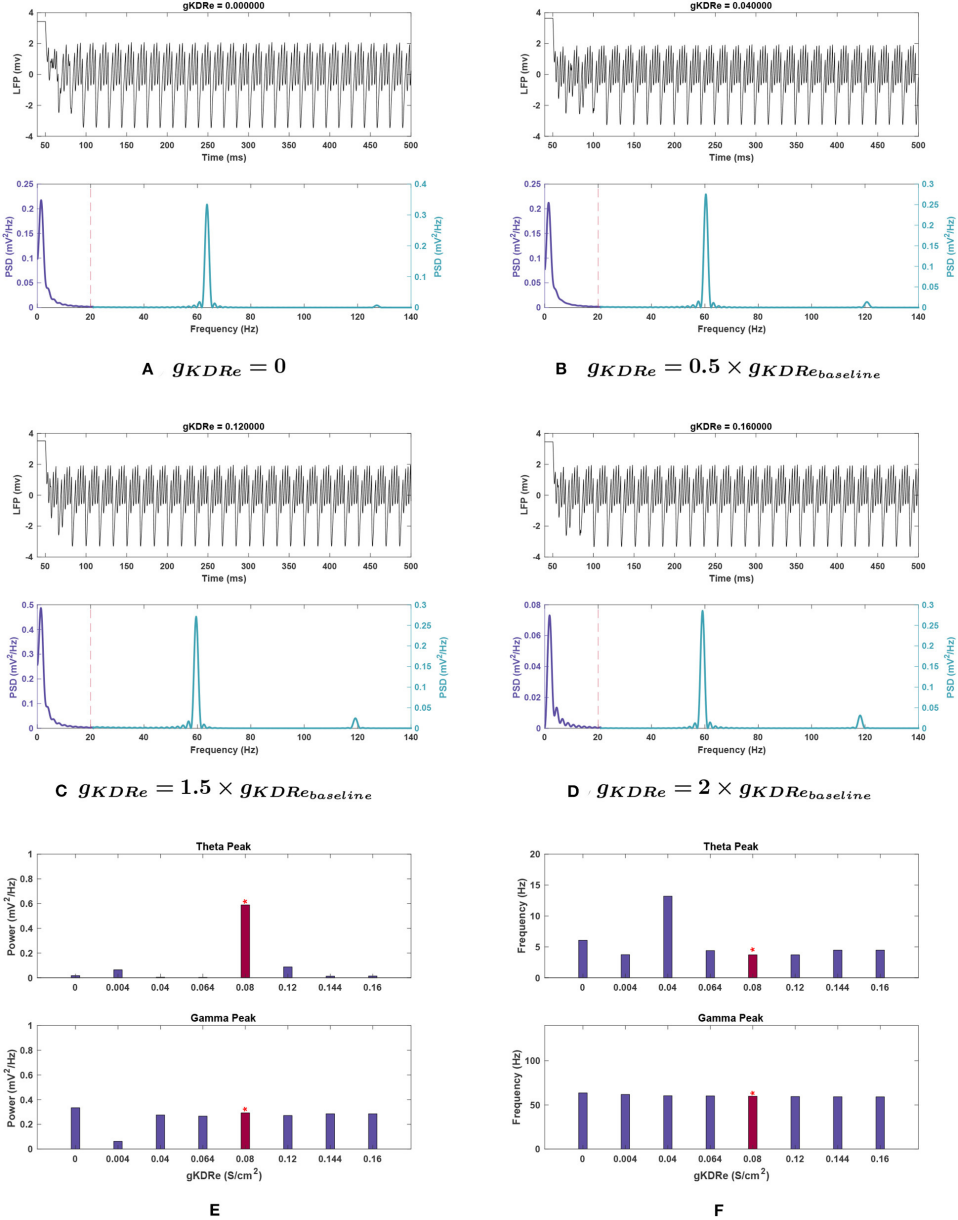
Impact of *g*_*KDRe*_ on network rhythmicity. **(A–D)** Graphs showing the network's LFP signal (top) and power spectrum (bottom) at selected conductances. **(E,F)** Bar plot showing changes in theta (top) and gamma (bottom) peaks in terms of **(E)** power and **(F)** frequency at varying values of *g*_*KDRe*_. Baseline value of *g*_*KDRe*_ and the corresponding results are represented with the red bars and the asterisk sign above them. Effects of varying *g*_*KDRe*_ show that the delayed rectifier potassium current in the E-cells have a very significant effect on theta rhythm generation, with its power greatly weakened in most cases. Gamma also appears to be reduced at very small conductance values for this current, but when the current is nullified altogether, the power rises above that seen in Figure 1.

**Figure 7:**
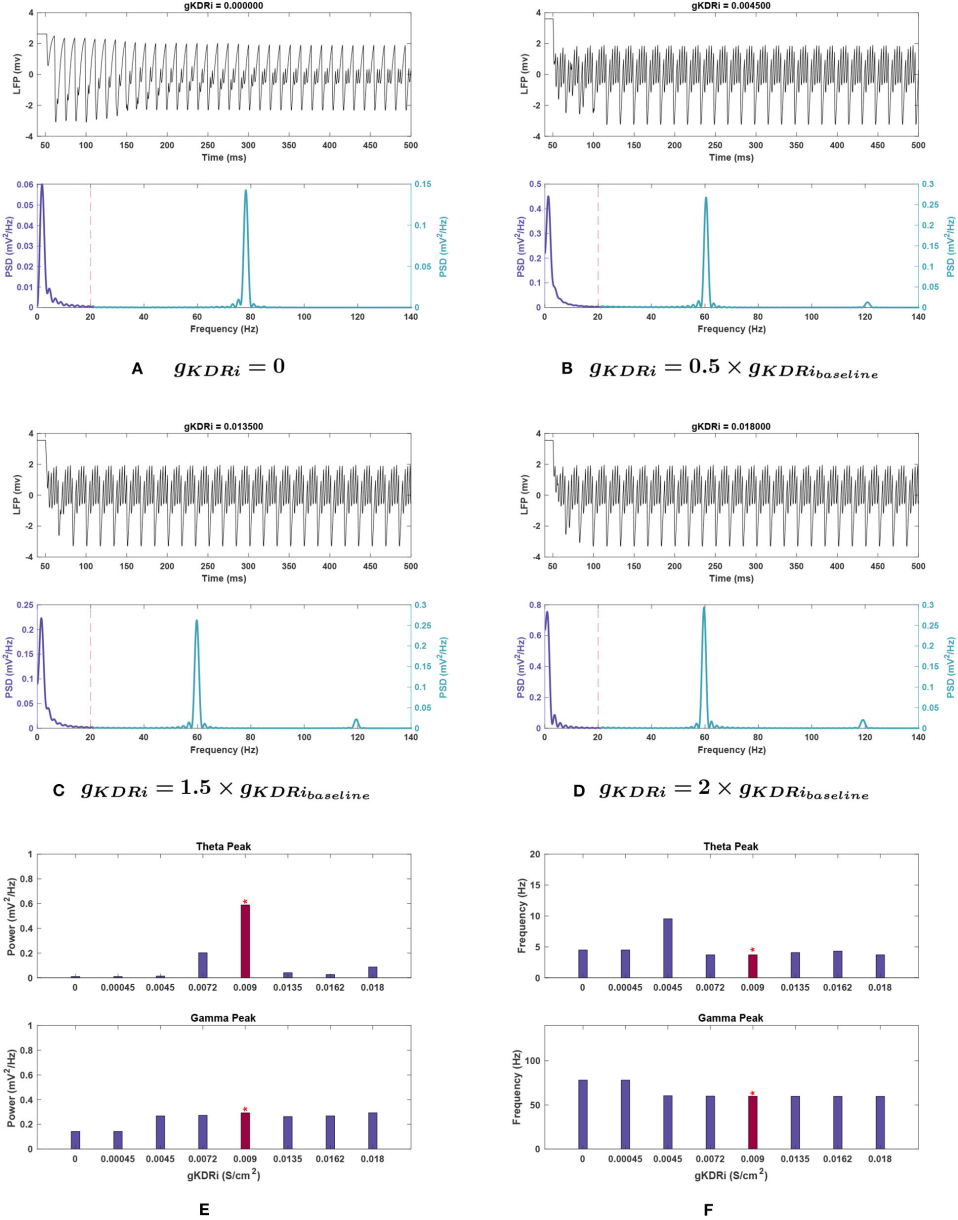
Impact of *g*_*KDRi*_ on network rhythmicity. **(A–D)** Graphic representation of network LFP signal (top) and power spectrum (bottom) for several selected *g*_*KDRi*_ values. **(E,F)** Fluctuations in **(E)** power and **(F)** frequency as seen in the theta (top) and gamma (bottom) bands in response to changing conductance. As mentioned before, the red bars with the asterisk sign denote the results at the baseline value of *g*_*KDRi*_. Similar to previous cases, the theta oscillations show a large decrease in power with any variation of *g*_*KDRi*_ when compared to the baseline conditions in Figure 1, and theta appears to be almost completely lost when this current is removed. *g*_*KDRi*_ has a more modest effect on gamma, but still notable; very small conductance values seem to reduce gamma power as well as push peak frequency higher.

**Figure 8:**
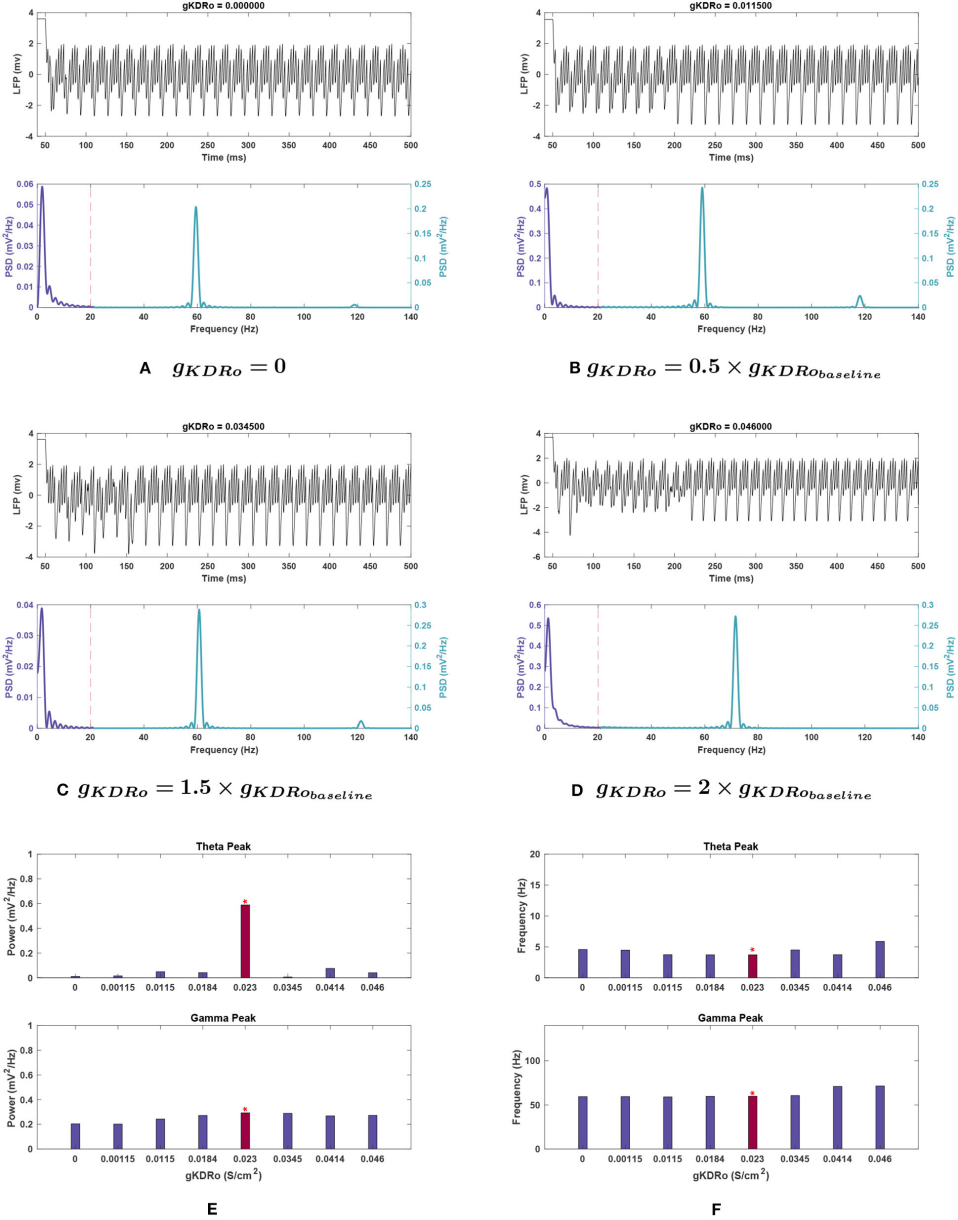
Impact of *g*_*KDRo*_ on network rhythmicity. **(A–D)** Changes in rhythmicity of model network shown through the LFP signal (top) and power spectra (bottom) at selected values of *g*_*KDRo*_. **(E,F)** Effects of modifying conductance on theta (top) and gamma (bottom) rhythms, in terms of **(E)** power and **(F)** frequency. Red bars with the asterisk sign are correspondent to the baseline values of *g*_*KDRo*_. The results suggest that changes in *g*_*KDRo*_ causes large variations in the theta range, with the power being reduced greatly in all cases compared to Figure 1. Gamma seems to be reduced at low values of this conductance, to a more moderate degree. In addition, it appears that the gamma peak starts being pushed toward higher frequencies at large increases in this current's conductance.

**Figure 9:**
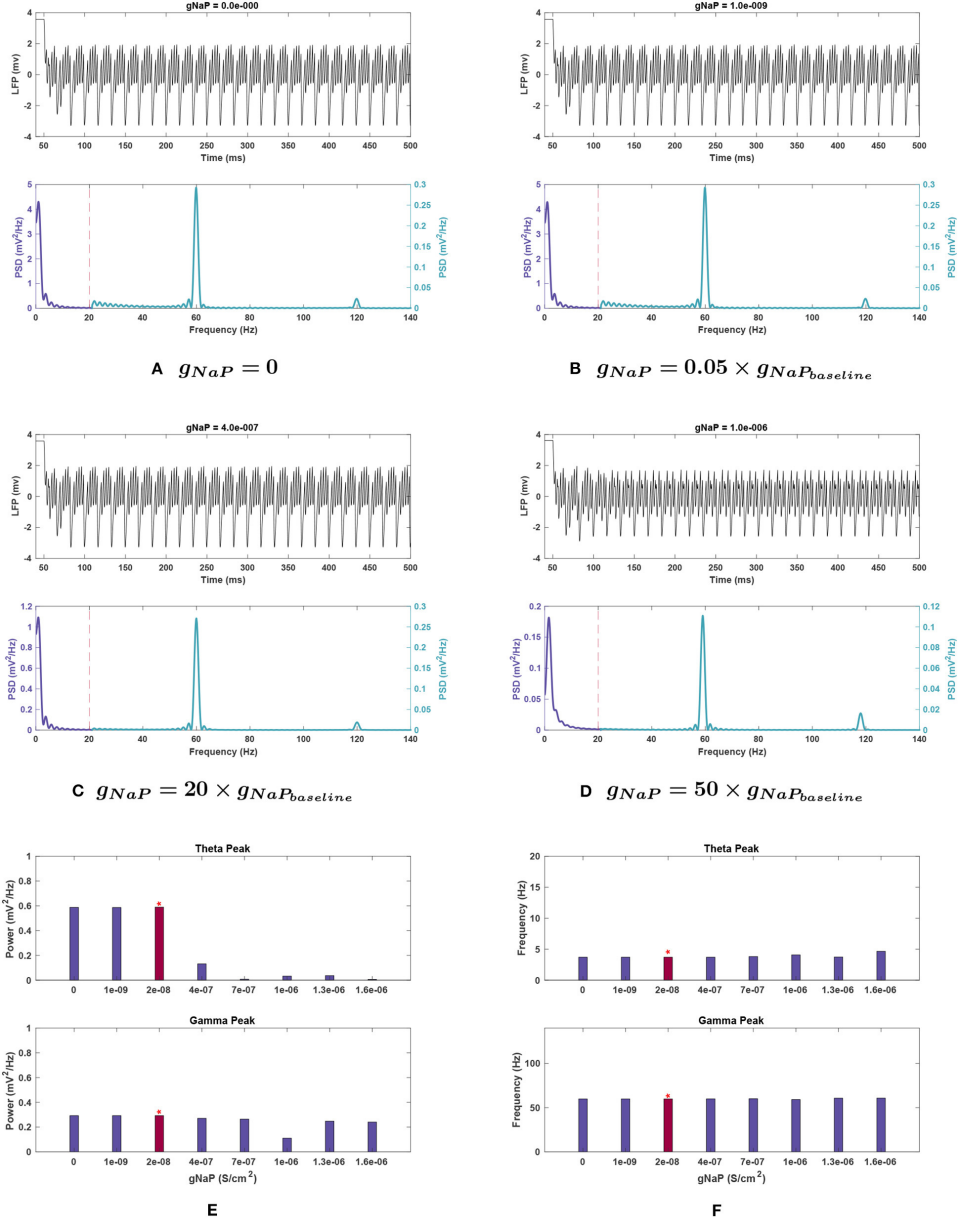
Impact of *g*_*NaP*_ on network rhythmicity. **(A–D)** Network LFP signal (top) and power spectra (bottom) observed at selected *g*_*NaP*_ values. **(E,F)** The values for **(E)** power and **(F)** frequency of peak theta (top) and gamma (bottom) network rhythms at varying *g*_*NaP*_. We have again used the red bars to highlight the results of the study with baseline *g*_*NaP*_ values. It is illustrated here that the increase in the persistent sodium current causes a significant reduction in theta band power. Gamma oscillations are weakened as well, though less substantially. Decreases in *g*_*NaP*_ do not appear to have any meaningful effect.

**Figure 10:**
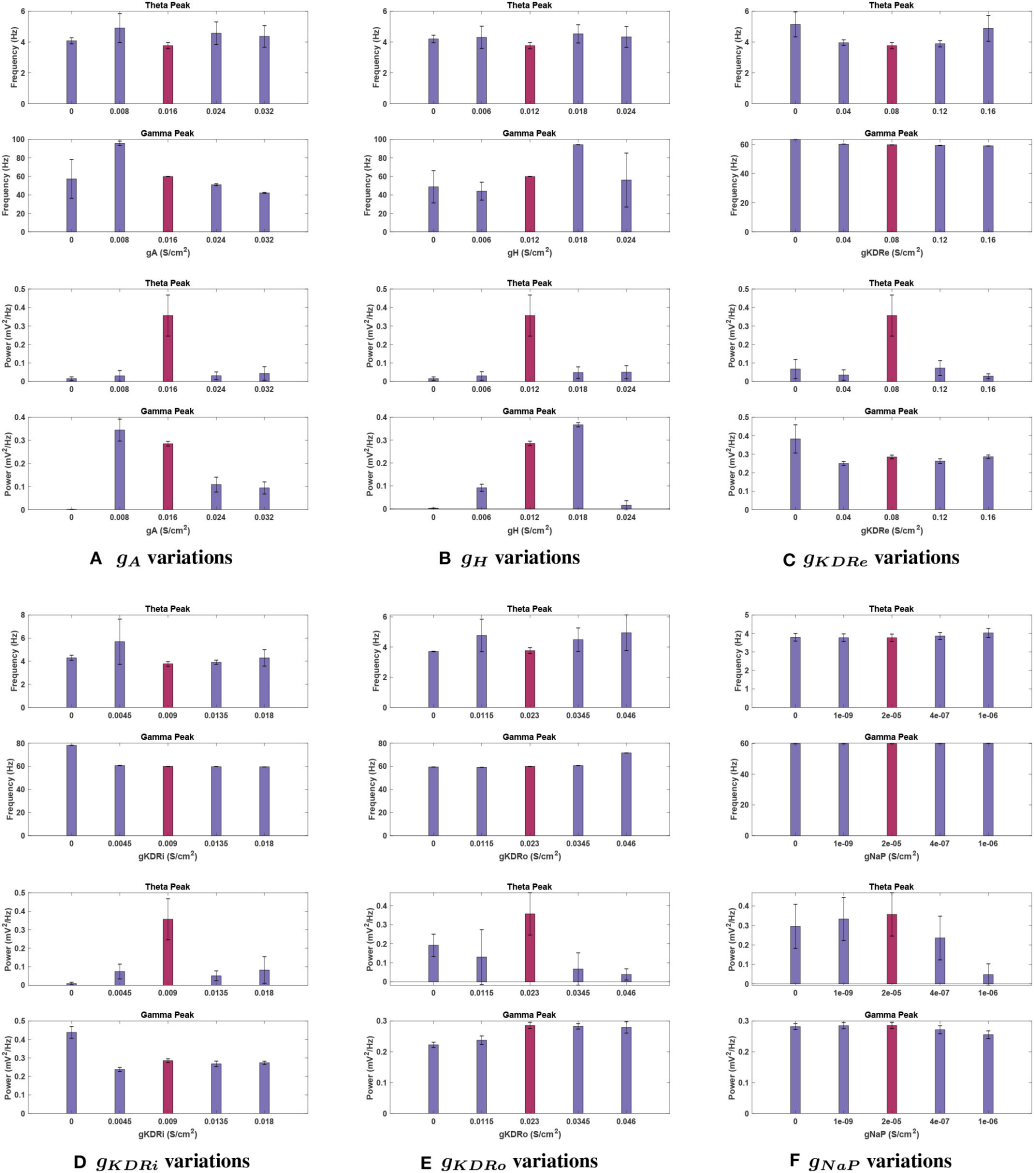
Effects of changing conductance values in a network containing noise. Graphs display the baseline conductance (red bars) and four adjusted conductance values for each current, **(A)** *g*_*A*_, **(B)** *g*_*H*_, **(C)** *g*_*KDRe*_, **(D)** *g*_*KDRi*_, **(E)** *g*_*KDRo*_, and **(F)** *g*_*NaP*_. Fluctuations in peak frequency (top) and power (bottom) are given at each conductance value, using 11 trials in a system containing noise to calculate average values. Bars display the mean, whereas vertical error lines are used to indicate the 95% confidence interval around the means in a *Student's t-test*. Notably, theta power and frequency appear to be much more sensitive to the existence of noise in the network than the gamma band, showing much greater variance in general.

The following sections report the results on LFP spectral properties resulting from adjusting the amplitude of each conductance separately. Figure 3 provides an overview of the variations in power spectrum in response to change of each of the 6 VGIC conductance values. In Figures 4–**9**, the effects of altering each conductance value is displayed separately. **Figure 10** shows the shifts in theta and gamma peak frequencies and power amplitudes, in response to modification of VGIC conductances with noise in the system.

#### 3.2.1. Effects of VGIC Modulation on Theta Rhythmicity

Theta rhythmicity was found to be affected significantly by changes in all VGIC conductances from baseline values. Generally, a decrease in the power of theta was observed and in some cases was completely abolished. Peak frequency shifts were also often observed.

***Effects of changingg*_*A*_**. Single trial runs modifying *g*_*A*_ showed that this conductance strongly influenced theta band power (see Figure 4E). Theta rapidly declined in power with decreasing *g*_*A*_, reducing by 98.2% when *g*_*A*_ was set to zero. The power was reduced to 4.3% of baseline value at 1.5 g, rose to 8.8% at 1.8 g, before being lost once again at 2 g with a reduction of 99.2%.

Theta peak frequency increased at 0.05, 1.5, and 2 g, rising by 21.7–24.7%, and remained stable at the remaining conductances (Figure 4E).

***Effects of changingg*_*H*_**. Theta power decreased steeply when *g*_*H*_ was reduced and was almost completely eliminated with a 98.3% reduction at 0.5 g. Further decreases in conductance caused a slight rebound of the peak's power to 16.4% of baseline. Raising *g*_*H*_ also caused theta power reduction, with a 30.5% decrease at 1.5 g, then being eliminated (99.5 and 99.4% reduction, respectively) at 1.5 and 2 g, its lowest points for this conductance (see Figure 5E).

The frequency of the theta peak remained largely stable throughout variations. At 0.8 and 1.8 g, where the peak frequencies shifted higher, the corresponding power was nearly abolished and hence, no significant peak was detected in the theta range (see Figures 5E,F). In these cases, the highest power content was identified in the delta range (1–3 Hz).

***Effects of changingg*_*KDR*_*in*** ***excitatory neurons (g*_*KDRe*_*)***. Figure 6E depicts that shifting *g*_*KDRe*_ in either direction from baseline causes a drastic decrease in theta band power. *g*_*KDRe*_ = 0.064 (0.8 g) and *g*_*KDRe*_ = 0.04 (0.5 g) displayed the greatest theta power reductions (99.5 and 99.1%, respectively).

At 0.8, 1.8, and 2 g, the peak frequency of theta increased by 18.6–21.7% (see Figure 6F). A large shift in peak frequency (by 197.9%) was recorded at 0.5 g, but theta power appeared totally suppressed at this conductance, so this was likely a composite peak between theta and gamma range rather than a peak shift.

***Effects of changingg*_*KDR*_*in*** ***interneurons (g*_*KDRi*_*)***. As seen in Figure 7E, theta power consistently declined with delayed rectifier conductance (*g*_*KDRi*_) reduction in interneurons. A large initial decrease of 65.8% was observed at 0.8 g, and theta was almost completely abolished as *g*_*KDRi*_ approached 0, with a 98.5% reduction in power. Initial increases in this conductance caused an even sharper reduction (93.0%), but at 2 g the power recovered to 14.7% of the baseline value.

The peak in theta frequency range shifted higher by 156.7% at 0.5 g but was associated with a large amplitude reduction of 97.7% (see Figure 7F). Other variations in *g*_*KDRi*_ had smaller effects causing either no shift (0.8, 2 g) or between 10.3 and 21.6% shift toward higher frequencies (0, 0.05, and 1.5 g).

***Effects of changingg*_*KDR*_*in oriens*** ***lacunosum interneurons (g*_*KDRo*_*)***. *g*_*KDRo*_ modulation in either direction from the baseline value significantly reduced theta power (Figure 8E). Decreases in conductance lowered theta power, with a 98.2% reduction at *g*_*KDRo*_ = 0.

Some variation in peak frequency was observed at 0, 0.05, and 1.5 g, but generally with reduced power (see Figure 8F).

***Effects of changingg*_*NaP*_**. For *g*_*NaP*_, there was no significant change in network activity in the theta band when conductance was decreased. This conductance was very robust to variations of a similar scale to those tested with the other conductances, so larger incremental steps were used. These larger changes reduced theta power, by 77.7% at *g*_*NaP*_ = 20 g, with even larger reductions at higher amplitudes (see Figures 9C,E).

Theta peak frequency was not strongly affected by *g*_*NaP*_ change (see Figure 9F) with a maximal increase of 25.7% observed at gNaP = 80 g.

#### 3.2.2. Effects of VGIC Modulation on Gamma Rhythmicity

Gamma rhythmicity was also found to be significantly modulated by alteration of VGIC conductances, though it was generally less sensitive than the theta band. In some cases, larger changes in conductance were required before the rhythm's stability was affected or more gradual changes were observed. Most effects observed were in the form of power decreases, sometimes leading to elimination of the rhythm, but at least one case was observed where power increased. Both increases and decreases in peak frequency were observed.

***Effects of changingg*_*A*_**. Gamma power tended to decrease with lowering *g*_*A*_. Notably, both gamma and higher frequency power were abolished when *g*_*A*_ was set to 0 (see Figure 4E). Like the theta band, gamma power is decreased but fluctuated as *g*_*A*_ increased (see Figure 4E).

Peak frequency shifted 19.8% higher at *g*_*A*_ = 0.8 g, and higher still (by 80.5%) at 0.5 g. However, it diminished greatly below 0.5 g and resulted in a decrease as high as 64.9% at 0.05 g, as seen in Figure 4F.

***Effects of changingg*_*H*_**. Gamma power consistently decreased with reduced *g*_*H*_, as seen in Figure 5E. The gamma power is almost abolished as *g*_*H*_ approaches 0 with 99% reduction from baseline power (see Figure 5A). Conversely, at *g*_*H*_ = 1.5 g, power increased by 38.1%, but the activity also shifted outside the typical gamma range to over 90 Hz (a 57.5% increase). Gamma power and frequency had opposing trends as *g*_*H*_ increased further. At 2 g, the power dropped significantly to 97.8% less than baseline (Figure 5D), but the peak continued rising to higher frequencies, reaching over 130 Hz, as shown in Figures 5E,F. While the detected peak frequencies at 1.8 and 2 g are outside the desirable range for gamma, the peak power is nearly abolished at these points which determines the insignificance of these disappearing peaks.

***Effects of changingg*_*KDR*_*in*** ***excitatory neurons (g*_*KDRe*_*)***. Gamma power displayed much less variation in *g*_*KDRe*_ trials, decreasing maximally by 8.4% in the trials ranging from 0.5 to 2 g. However, at 0.05 g, gamma power dropped abruptly by 78.8% of baseline value. At *g*_*KDRe*_ = 0, the power then increased to 14.2% above baseline value (see Figures 6A,E, respectively).

The gamma peak frequency was generally stable with a maximal 6.2% increase at *g*_*KDRe*_ = 0 and shifts of <4% otherwise (see Figure 6F).

***Effects of changingg*_*KDR*_*in*** ***interneurons (g*_*KDRi*_*)***. Like in the E-cells, gamma power was mostly stable in *g*_*KDRi*_ trials as seen in Figure 7E. The peak power was reduced by 10.3% of the baseline value at 1.5 g, and less variation was observed in most other instances. This deviated at *g*_*KDRi*_ = 0 and *g*_*KDRi*_ = 0.05 g, where the power dropped by 50.5%, indicating a significant role of this conductance in gamma rhythmogenesis.

In addition, gamma peak frequency was increased 30.5% to nearly 80 Hz at *g*_*KDRi*_ = 0 and *g*_*KDRi*_ = 0.05 g (Figures 7A,F). The high frequency peak normally present at 120 Hz was abolished in these trials.

***Effects of changingg*_*KDR*_*in oriens*** ***lacunosum interneurons (g*_*KDRo*_*)***. Gamma behaved similarly in *g*_*KDRo*_ and *g*_*KDRi*_, though a steady downward trend in power was observed both when increasing and decreasing *g*_*KDRo*_. The power reduction did not exceed 20% of the baseline until 0.05 g, where the power dropped by 30.9%.

Gamma peak frequency only shifted at 1.8 and 2 g, where it was increased by 18.6 and 19.4%, respectively. 1.8 and 2 g were also the two conductance values where loss of the high-frequency peak at 120 Hz occurred (see Figure 3D).

***Effects of changingg*_*NaP*_**. As with theta, reduced amplitude of *g*_*NaP*_ compared to baseline had no appreciable effect on gamma rhythmicity. Increased *g*_*NaP*_ gradually decreased gamma power, with the greatest effect at *g*_*NaP*_ = 50 g (see Figure 9D), where the gamma peak's power abruptly dipped to 38.0% of baseline conditions.

Gamma peak frequency remained stable with variations in this conductance (Figure 9F).

### 3.3. Effects of Noise on Network Response to VGIC Modulation

In this section, we employ the simulated model with added noise to depict how VGIC conductance variations can affect this system.

#### 3.3.1. Effects of Noise With VGIC Modulation on Theta Rhythmicity

Though many of the rhythmicity trends observed with noise were comparable to our noiseless trials, theta displayed significant sensitivity to this additional factor being introduced into the system. Theta power was more affected by the addition of noise than gamma and continued to be more affected by conductance modulation as well, consistent with the noiseless trials. The results in this section are obtained using 11 trials and reported as (*mean* ± *SEM*) in a *Student's t-test*.

***Effects of changingg*_*A*_**. Theta power reduction was consistent with noiseless runs with respect to *g*_*A*_, with a key difference at 2 g. This *g*_*A*_ value had the highest mean power for theta among *g*_*A*_ trials, not counting the baseline case (0.043 ± 0.036 $\frac{mV^{2}}{Hz}$); theta was functionally lost at this conductance without noise (see Figure 10A). Theta power still became negligibly small (0.015 ± 0.009 $\frac{mV^{2}}{Hz}$) with *g*_*A*_ = 0, reinforcing the significant role of this current in theta rhythmogenesis.

Theta peak frequency was shifted upwards for all variations of *g*_*A*_ moving from 3.70 Hz at baseline to its highest point of 4.907 ± 0.932 Hz at 0.5 g.

***Effects of changingg*_*H*_**. When examining *g*_*H*_ modulation with noise (see Figure 10B), the most significant differences were in theta power. While the theta peak was almost completely nullified at 0.5 and 2 g without noise, with added noise the theta power followed an increasing trend in moving from 0 until 2 g. Nevertheless, compared to the baseline power observed with noise, 0.356 ± 0.111 $\frac{mV^{2}}{Hz}$, it dropped significantly for all these variations of *g*_*H*_ (compare Figures 5E, 10B). The lowest mean power was at *g*_*H*_ = 0 (0.015 ± 0.009 $\frac{mV^{2}}{Hz}$) but great variability was observed in this simulation, with the *SEM* amplitude comparable to the mean value (±0.035 $\frac{mV^{2}}{Hz}$).

Mean theta peak frequency was elevated (13.5–22.4%) compared to the baseline in all four *g*_*H*_ modulations tested.

***Effects of changingg*_*KDR*_*in*** ***excitatory neurons (g*_*KDRe*_*)***. For *g*_*KDRe*_ (see Figure 10C), theta power trends remained similar to those in the noiseless system; theta was greatly reduced from the baseline condition in all cases, down to 0.029 ± 0.012 $\frac{mV^{2}}{Hz}$ at 2 g. However, the *SEM* values at 0, 0.5, 1.5, and 2 g are all large compared to the mean (±0.052, ±0.028, ±0.040, and ±0.012 $\frac{mV^{2}}{Hz}$, respectively).

Theta frequency remained relatively stable among *g*_*KDRe*_ values, lacking the large shift seen in the noiseless case at 0.5 g (compare Figures 6F, 10C).

***Effects of changingg*_*KDR*_*in*** ***interneurons (g*_*KDRi*_*)***. Mean theta power remained consistent for most *g*_*KDRi*_ cases with noise compared to noiseless runs (compare Figures 7E, 10D), showing clear decreased values for all variations of *g*_*KDRi*_. The notable exception was 0.5 g where without noise, the theta peak was lost. With added noise, it remained present, while still greatly reduced from 0.356 ± 0.111 $\frac{mV^{2}}{Hz}$ at baseline to 0.074 ± 0.040 $\frac{mV^{2}}{Hz}$. Although the mean theta power at 2 g was similar to the noiseless equivalent, it displayed significant run to run variability with a large *SEM* (0.082 ± 0.072 $\frac{mV^{2}}{Hz}$).

Theta frequency showed deviations at 0.5 and 2 g. The mean frequency did not increase as markedly as noiseless experiments at 0.5 g and had a very large *SEM* (5.687 ± 1.957 $\frac{mV^{2}}{Hz}$). An increase in peak theta frequency from 3.766 ± 0.198 Hz at baseline to 4.286 ± 0.711 Hz at 2 g was also observed, unlike noiseless trials.

***Effects of changingg*_*KDR*_*in oriens*** ***lacunosum interneurons (g*_*KDRo*_*)***. The addition of noise led to significant deviation in the theta power band with *g*_*KDRo*_ variations (see Figure 10E). Though the mean power at 2 g was consistent with noiseless runs, trials at 0, 0.5, and 1.5 g had mean power significantly above their equivalents in Figure 8E. This was particularly apparent at *g*_*KDRo*_ = 0 and 1.5 g, where the theta peak was abolished in noiseless runs. With noise, *g*_*KDRo*_ = 0 had the highest power in this band (0.192 ± 0.058 $\frac{mV^{2}}{Hz}$) outside of the baseline conductance. Theta power trends seemed to lower with increasing *g*_*KDRo*_; conversely, after an initial reduction, power increased with decreasing *g*_*KDRo*_. However, the reported standard error values were very large, particularly at 0.5 and 1.5 g, where theta power was 0.130 ± 0.143 and 0.067 ± 0.085, respectively, vs. 0.356 ± 0.111 $\frac{mV^{2}}{Hz}$ at baseline. This indicates the broad disparity among the trials leading to occasional elimination of theta peak at some trials of 0.5 and 1.5 g.

Theta frequency mostly followed a similar trend in the noiseless and noisy cases. However, 0.5 g had an increased mean frequency (4.768 Hz) in this band compared to the baseline value, and *g*_*KDRo*_ = 0 was unchanged from baseline frequency. These both differ from the noiseless results in Figure 8F. Most theta frequency values had a wide confidence interval between ±0.776 and ±1.178 $\frac{mV^{2}}{Hz}$, except *g*_*KDRo*_ = 0 (3.707 ± 0.015 $\frac{mV^{2}}{Hz}$).

***Effects of changingg*_*NaP*_**. *g*_*NaP*_ effects with noise are displayed in Figure 10F. As with other conductances, the largest deviations were in theta power (compare with Figure 9E). Interestingly, though no change was observed in noiseless trials when *g*_*NaP*_ was decreased, the mean power decreased when doing the same with noise, going to 0.235 ± 0.112 $\frac{mV^{2}}{Hz}$ at 20 g and as low as 0.048 ± 0.054 $\frac{mV^{2}}{Hz}$ at 50 g. The latter implies that peak theta power would be completely diminished on the lower end of the confidence interval and the disparity levels are significantly high in the *g*_*NaP*_ = 50 g trials. Further increasing *g*_*NaP*_ still decreased the overall theta power. Nevertheless, the initial decrease observed at *g*_*NaP*_ = 20 g was no longer as steep, the mean theta power only being reduced by 34.0% of baseline power. The *SEM* is considerably large compared to the mean (0.235 ± 0.112 $\frac{mV^{2}}{Hz}$).

The peak frequency at theta range stayed highly stable, similar to noiseless runs, with its largest change being from 3.766 ± 0.198 Hz at baseline to 4.026 ± 0.240 Hz at *g*_*NaP*_ = 80 g.

#### 3.3.2. Effects of Noise With VGIC Modulation on Gamma Rhythmicity

Introducing noise to the system had significant effects on several cases of VGIC modulation for the gamma band. However, many trends remained very similar or identical to those observed in the absence of noise. In most cases, the gamma power and frequency displayed smaller and more uniform *SEM* values than theta. Overall, this rhythm appeared very robust to the addition of noise into the network.

***Effects of changingg*_*A*_**. The introduction of noise interacted significantly with *g*_*A*_ amplitude effects on gamma power as shown in Figure 10A. For example, at 0.5 g, gamma power was reduced by 76.2% without noise, but with noise there was an increase of 20.7% of the mean power. At *g*_*A*_ = 0, gamma power was absent, while after that increasing *g*_*A*_ values up to 2 g reduced gamma power.

Gamma peak frequency showed very little variance across trials and was consistent with noiseless trials; the only exception was for *g*_*A*_= 0, but this was more likely an effect of the peak being negligibly small rather than a significant shift. Interestingly, 0.5 g resulted in a greatly increased peak frequency (95.797 ± 2.436 vs. 59.910 ± 0.033 Hz at baseline), which matched the noiseless run.

***Effects of changingg*_*H*_**. Gamma peak power followed the same trend as seen in standard conditions for *g*_*H*_ (compare Figure 10B with Figure 5E). This includes the power increase at 1.5 g, and the loss of gamma at *g*_*H*_ = 0. However, the power at 0.5 g was greater than corresponding noiseless conditions (0.092 ± 0.015 $\frac{mV^{2}}{Hz}$). Conversely, power at 2 g was considerably smaller than the noiseless run, measuring 0.015 ± 0.020 $\frac{mV^{2}}{Hz}$—the peak would be completely lost on the lower end of the confidence interval.

The peak frequency for 2 g showed a very wide error margin, and the mean was lower than baseline value (56.080 ± 29.190 vs. 59.910 ± 0.033 Hz). This was in contrary to the standard run, where this conductance value measured the highest frequency gamma peak. The other conductances tested had similar peaks to the noiseless run, with 1.5 g being particularly resistant to variability. However, the mean frequency at *g*_*H*_ = 0 was higher than that of 0.5 g under noisy conditions (48.740 vs. 44.031 Hz), opposite to the noiseless network. *g*_*H*_ = 0 also showed a large *SEM* in frequency (±17.432 Hz), but as the peak was functionally absent at this conductance, this variability is less likely to be an effect of network noise.

***Effects of changingg*_*KDR*_*in*** ***excitatory neurons (g*_*KDRe*_*)***. Both gamma power and frequency trends remained nearly identical to standard noiseless simulations for *g*_*KDRe*_. The gamma band showed much less fluctuation across noise trials than theta and seemed especially resistant to variation in peak frequency. A notable difference is at *g*_*KDRe*_ = 0 where despite gamma power still increasing, the mean (0.383 ± 0.076 $\frac{mV^{2}}{Hz}$) was higher than the noiseless value (compare with Figure 6E).

***Effects of changingg*_*KDR*_*in*** ***interneurons (g*_*KDRi*_*)***. For *g*_*KDRi*_, gamma had mean power and peak frequency values and trends identical to those seen without noise, with only one significant deviation at *g*_*KDRi*_ = 0. Rather than being reduced, the mean gamma power observed was significantly higher than the baseline value (0.438 vs. 0.285 $\frac{mV^{2}}{Hz}$), while the peak frequency remained consistent.

***Effects of changingg*_*KDR*_*in oriens*** ***lacunosum interneurons (g*_*KDRo*_*)***. Like the I-cell counterpart, gamma band frequency and power under *g*_*KDRo*_ modulation were nearly identical to noiseless trials and followed the same trends.

***Effects of changingg*_*NaP*_**. The same general trends were observed in gamma power as the noiseless runs for *g*_*NaP*_, but a significant deviation occurred at *g*_*NaP*_ = 50 g. The sharp decrease in peak gamma power at this conductance was no longer observed, as the mean power instead decreased more gradually, by 10.5% of the baseline power.

Gamma peak frequencies were mostly consistent with the noise-free simulations, except for the gamma peak at *g*_*NaP*_ = 50 g, which no longer decreased from baseline frequency.

## 4. Discussion

We have simulated a PING network of pyramidal excitatory neurons, inhibitory interneurons, and oriens lacunosum moleculare interneurons. Subsequently, we have examined the effects of varying VGIC conductance through multiple simulation runs. These experiments were then repeated with noise applied to the E-drive used in the model in order to examine the effect of random noise on these rhythms and to simulate more realistic network conditions. Apart from improving understanding of the fundamental mechanisms of rhythmogenesis in neural networks, these observations are also of interest as many commonly used psychoactive drugs are known to modulate voltage-gated ion channels either directly (e.g., phenytoin and lamotrigine on Nav channels) or indirectly (e.g., SSRIs on Nav and Kv channels and antipsychotic drugs on Kv class channels).

The results in section 3 show that theta rhythmicity is very responsive to changes in VGIC conductance values. The most widely observed effects of conductance modulation were substantial decreases in the power of the theta rhythm and, in many cases, resulting in loss of the peak. There were no observed cases where the power of the theta peak increased compared to baseline. Under noise-free conditions, absence of *I*_*A*_ or any of the *I*_*KDR*_ currents lead to loss of theta peak, suggesting a likely dependence of the theta peak on these currents. Conversely, relatively high conductance in *I*_*A*_, *I*_*H*_, *I*_*KDRe*_, or *I*_*NaP*_ resulted in loss of this peak. In light of these findings, introduction of noise appeared to prevent theta loss at high *I*_*H*_, low *I*_*KDRo*_, and both high and low *I*_*KDRe*_. Additionally, removing *I*_*H*_ in a noisy network resulted in theta loss instead. The generally large amounts of variance observed across all the trials containing noise suggest that this rhythm may also be highly sensitive to other network conditions as well as VGIC conductance shifts.

Gamma band oscillations were also significantly affected by variation in VGIC conductances. Gamma rhythmicity showed less susceptibility to changing conductance compared to the theta band. Most notably, the gamma band disappeared when the *I*_*H*_ and *I*_*A*_ currents were set to 0. This implies a likely dependence on these currents for the gamma rhythm to be generated. Specifically, *I*_*H*_ and *I*_*A*_ seemed to have inverse effects on gamma. Increasing the *H*-current increased gamma power at first, but this trend was reversed at higher conductances. Conversely, decreasing the *A*-current had little effect on gamma rhythm at low amplitude changes, but weakened it steeply as the conductance declined further. *I*_*KDRi*_ and *I*_*KDRe*_ currents demonstrated similar trends whereby gamma power declined with decreasing *g* values. This trend was not seen in the E-cells, where the absence of *I*_*KDRe*_ enhanced gamma. This could imply that the current poses a regulatory effect in maintaining gamma within a certain range. *I*_*NaP*_ generally decreased the gamma peak as it was elevated, though the extent of the decrease fluctuated. The addition of noise demonstrated less impact on gamma rhythmicity compared to the theta band. Nevertheless, some noteworthy shifts still occurred. Primarily, the *I*_*KDRi*_ current showed the same trend as its E-cell counterpart with noise, whereas removal of the current resulted in gamma being amplified. Additionally, decreasing *g*_*A*_ under noisy conditions increased gamma power at first. Further decreases in conductance followed the same trend as before, with the peak being lost following removal of the current. Hence, the inverse correlation of *I*_*A*_ and *I*_*H*_ seems even more apparent under noisy conditions.

Our findings demonstrate a strong association between voltage-gated ion channel conductance and network rhythmicity. Few studies have examined this phenomenon and covered the wide range of VGICs as accomplished in this work. Wang (1993) investigated persistent sodium and slowly inactivating potassium currents and examined how the presence of their interplay produces oscillations in the 10–50 Hz range. Our findings differ from this, as we did not observe any significant decrease in gamma activity with lowered *g*_*NaP*_. Instead, we saw a decrease in theta power following introduction of noise. The Wang model contained fewer currents and observed a single cell environment, so it is unclear if this deviation can simply be attributed to differences in simulation conditions. Another study by Xu et al. (2004) showed that the use of a targeted *H*-current blocker on septo-hippocampal neurons *in vivo* resulted in a decrease in hippocampal theta rhythm. Though the range at which they observed their theta peak (5–8 Hz) differs from that seen in our model, we also observed a decrease in theta when lowering *g*_*H*_. In the absence of other clear analogs, it otherwise appears that the observations seen in our simulations are novel findings requiring further investigation. Abnormalities of neural network rhythmicity have been associated with several disorders of the brain. Alzheimer's disease has been associated with a decrease in gamma activity (Herrmann and Demiralp, 2005; Mably and Colgin, 2018) and an increase in theta (Bhattacharya et al., 2011; Vecchio et al., 2013); both phenomena are associated with cognitive deficits. Gamma band irregularities have also been implicated in epilepsy (Herrmann and Demiralp, 2005), schizophrenia (Herrmann and Demiralp, 2005; Roopun et al., 2008; Uhlhaas and Singer, 2015) and depression (Fitzgerald and Watson, 2018). The relevance of network oscillations is further corroborated by research connecting them to memory and learning (Yamamoto et al., 2014). Theta and gamma oscillations, as well as theta–gamma coupling, are believed to be differentially modulated during the learning phase (Priori et al., 2004).

Furthermore, many pharmacological treatments for neurodegenerative disorders have known relevant effects on network rhythmicity. Dopaminergic drugs are found to enhance theta rhythms in Parkinson's disease (Priori et al., 2004). Antipsychotic drugs reduce abnormally high gamma in schizophrenia (Ahnaou et al., 2017). Acetylcholinesterase inhibitors and the NMDA antagonist memantine are cognition-enhancing drugs used in Alzheimer's treatment; they have been found to increase both theta and gamma rhythmicity in rats (Guadagna et al., 2012). Interestingly, some treatment methods have shown effects on VGICs but have not been linked with any observed abnormal rhythms. Inhibition of *I*_*Na*_ and *I*_*NaP*_ are well-established as mechanisms of epilepsy treatment (Köhling, 2002; Ahnaou et al., 2014). Potassium channels are also speculated to have a significant role in epilepsy (Errington et al., 2005), as some anti-convulsives seem to work primarily through potassium channel modulation (Köhling, 2002; Wickenden, 2002). Acetylcholinesterase inhibitors have also been found to inhibit both *Na*^+^ and *K*^+^ VGICs (Gunthorpe et al., 2012).

This study reveals strong modulatory effects of several classes of VGICs on functionally significant neural network oscillations in the utilized PING network model. It will be of interest to explore pharmacological blockade of these channels *in vivo*, in association with cognitive performance. These results also raise the question whether some commonly used drugs with VGIC targets, such as antipsychotics and antiepileptics, may also target network frequency modulation. Further computational or *in vivo* studies are required to better characterize and corroborate the effects *I*_*A*_, *I*_*H*_, *I*_*KDRe*_, *I*_*KDRi*_, *I*_*KDRo*_, and *I*_*NaP*_ currents have on neural oscillations. Investigating all these parameters in the hippocampal neural network model leads to a very broad exploring space. While we demonstrate in Supplementary Material that manipulating the drive currents (specifically *I*_*E*_ and *I*_*I*_) visibly affects the operating regime and the oscillations of the network, we leave further investigation of these effects to future works. Another consideration for future studies would be to experiment with co-varying VGIC conductances and observing their effects on VGICs, as co-variance has been explored successfully in other work (Ratté et al., 2014). This would further characterize the role of particular VGICs in network rhythmicity as well as their relationship to each other.

The current model successfully reproduces theta and gamma oscillations and is based on plausible physiological data. To the best of our knowledge, the Kopell model is among the very few studies which enable us to study the effects of several voltage gated ion channels. It is also shown that there is concordance between biological phenomena and the results of this model (Scheffer-Teixeira and Tort, 2016; Jansen et al., 2021). Nevertheless, there are also limitations to this model. For instance, alternate regimes can be considered for future iterations of this model or creation of new models which benefit from more realistic pyramidal neuron interconnectivity (Thomson and Bannister, 2003; Douglas and Martin, 2004) and alternative firing patterns (Moca et al., 2014). These are among the elements which were not included in this study for simplicity. Moreover, to avoid excess complexity of the model, this work does not incorporate other important components, such as the three types of glial cells. It has been well-established that all three types of glial cells are important for memory formation and learning (Tewari and Parpura, 2013, 2016; Sibille et al., 2014; Verkhratsky et al., 2015; Gibbs, 2016; Tewari et al., 2016). Therefore, an augmented version of the present model which encompasses other components of the hippocampal region remains to be investigated in the future works. This could lead to a better understanding of all the important and role-playing elements in modulating the local field potential rhythms. Expanding our understanding of all these interactions will likely lead to novel treatments of disorders characterized by neural rhythmic abnormalities.

## Data Availability Statement

The original contributions presented in the study are included in the article/Supplementary Material, further inquiries can be directed to the corresponding author/s.

## Author Contributions

CF defined the project. CO and FH conceived and designed the experiments, performed the experiments, analyzed the data, contributed the reagents, materials, analysis tools, and wrote the paper. All authors reviewed the manuscript.

## Conflict of Interest

The authors declare that the research was conducted in the absence of any commercial or financial relationships that could be construed as a potential conflict of interest.
