# Supplementary material for: Effects of Several Classes of Voltage-Gated Ion Channel Conductances on Gamma and Theta Oscillations in a Hippocampal Microcircuit Model
Source: Front Comput Neurosci. 2021 Apr 1;15:630271. doi: 10.3389/fncom.2021.630271 (PMC8049632; doi:10.3389/fncom.2021.630271)
Supplement: Supplementary file 1 [file Presentation_1.pdf]

# Effects of Several Classes of Voltage-Gated Ion Channel Conductances on Gamma and Theta Oscillations in a Hippocampal Microcircuit Model

**Chris Olteanu [1*], Forough Habibollahi [1,2*], and Chris French [3]**

[1] *Melbourne Brain Centre, The University of Melbourne, Victoria, Australia*
[2] *Department of Biomedical Engineering, The University of Melbourne, Victoria, Australia*
[3] *Department of Medicine, The Royal Melbourne Hospital, The University of Melbourne, Victoria, Australia*

**Correspondence:**
**Chris French**
**frenchc@unimelb.edu.au**

## SUPPLEMENTARY MATERIALS

In this section, we will introduce the mathematical details of the utilized model and introduce the equations and parameters that derive the PING network in this work.

## 1 NEURON MODELS

We use the following models for each type of the neurons in our study:

- **E-Cells:** Based on the model of Olufsen et al. (2003) and extending it to incorporate the persistent sodium conductance ($g_{NaP}$) of the E-Cells as well, we use:

$$C\frac{dV}{dt} = g_{Na}m_{\infty}V^3h(V_{Na}-V)+g_kn^4(V_k-V)+g_L(V_L-V)+g_{NaP}r_{\infty}(V_{NaP}-V)+I \quad (1)$$

$$\frac{dh}{dt} = \frac{h_{\infty}(V)-h}{\tau_h(V)} \quad (2)$$

$$\frac{dn}{dt} = \frac{n_{\infty}(V)-n}{\tau_n(V)} \quad (3)$$

where we have:

$$x_{\infty}(V) = \frac{\alpha_x(V)}{\alpha_x(V)+\beta_x(V)} \qquad for \;\; x = m, h, or \;\; n \quad (4)$$

*Both authors contributed equally to this manuscript.

and

$$\tau_x(V) = \frac{1}{\alpha_x(V) + \beta_x(V)} \qquad for \;\; x = h \;\; or \;\; n \tag{5}$$

In the above equations, the letters $C$, $V$, $t$ and $\tau$, $g$, and $I$ denote capacitance density, voltage, time, conductance density, and current density, respectively. The corresponding units for these quantities are $\frac{\mu F}{cm}$ , $mV$, $ms$, $\frac{mS}{cm}$ , and $\frac{\mu A}{cm}$. For this part of the model, we use the following parameters: $C = 1, g_{Na} = 100, g_K = 80, g_L = 0.1, V_{Na} = 50, V_K = -100, V_L = -67$.
Finally, for the $\alpha_x$ and $\beta_x$ parameters we have:

$$\begin{aligned}
\alpha_m(V) &= \frac{0.32(V+54)}{1 - exp(-(V+54)/4)} \\
\beta_m(V) &= \frac{0.28(V+27)}{exp((V+27)/5) - 1} \\
\alpha_h(V) &= 0.128 exp(-(V+50)/18) \\
\beta_h(V) &= \frac{4}{1 + exp(-(V+27)/5)} \\
\alpha_n(V) &= \frac{0.032(V+52)}{1 - exp(-(V+52)/5)} \\
\beta_n(V) &= 0.5 exp(-(V+57)/40)
\end{aligned} \tag{6}$$

Finally, with the addition of persistent sodium conductance based on Li and Cleland (2013), we have:

$$r_\infty = \frac{1}{1 + exp(-(V+50)/5)} \tag{7}$$

- **I-Cells:** Based on Wang and Buzsáki (1996), we follow the below equations for modeling the I-cells in this work:

$$C\frac{dV}{dt} = g_{Na} m_\infty V^3 h(V_{Na} - V) + g_k n^4 (V_k - V) + g_L(V_L - V) + I \tag{8}$$

$$\frac{dh}{dt} = \frac{h_\infty(V) - h}{\tau_h(V)} \tag{9}$$

$$\frac{dn}{dt} = \frac{n_\infty(V) - n}{\tau_n(V)} \tag{10}$$

where we have:

$$x_\infty(V) = \frac{\alpha_x(V)}{\alpha_x(V) + \beta_x(V)} \qquad for \;\; x = m, h, or \;\; n \tag{11}$$

and

$$\tau_x(V) = \frac{0.2}{\alpha_x(V) + \beta_x(V)} \qquad for \;\; x = hor \;\; n \tag{12}$$

where we have the rate functions as the following:

$$\begin{aligned}\alpha_m(V) &= \frac{0.1(V+35)}{1-exp(-(V+35)/10)}\\ \beta_m(V) &= 4exp(-(V+60)/18)\\ \alpha_h(V) &= 0.07exp(-(V+58)/20)\\ \beta_h(V) &= \frac{1}{exp(-0.1(V+28))+1}\\ \alpha_n(V) &= \frac{0.01(V+34)}{1-exp(-0.1(V+34))}\\ \beta_n(V) &= 0.125exp(-(V+44)/80)\end{aligned} \tag{13}$$

The parameters utilized, are in the same units as for the E-cells and we have $C = 1, g_{Na} = 35, g_K = 9, g_L = 0.1, V_{Na} = 55, V_K = -90, V_L = -65$.

- **O-Cells:** We refer to the model of Tort et al. (2007) for defining the O-cells which is itself a reduction of the multi-compartmental model in Saraga et al. (2003). Therefore, we get:

$$C\frac{dV}{dt} = g_{Na}m^3h(V_{Na}-V) + g_kn^4(V_k-V) + g_Aab(V_A-V) + g_hr(V_h-V)g_L(V_L-V) + I \tag{14}$$

with

$$\frac{dx}{dt} = \frac{x_\infty(V)-x}{\tau_x(V)} \qquad for\ \ x = m,h,n,a,b,r \tag{15}$$

For $x = m, n, h$ the functions $x_\infty(V)$ and $\tau_x(V)$ are the same as in Equations 4 and 5, and now the rate functions can be defined as follows:

$$\begin{aligned}\alpha_m(V) &= \frac{0.1(V+38)}{exp(-(V+38)/10)-1}\\ \beta_m(V) &= 4exp(-(V+65)/18)\\ \alpha_h(V) &= 0.07exp(-(V+63)/20)\\ \beta_h(V) &= \frac{1}{exp(-(V+33)/10)+1}\\ \alpha_n(V) &= \frac{0.018(V-25)}{1-exp(-(V-25)/25)}\\ \beta_n(V) &= \frac{0.0036(V-35)}{exp((V-35)/12)-1}\end{aligned} \tag{16}$$

Eventually, for $x = a, b, r$, we present the $x_\infty(V)$ and $\tau_x(V)$ as below:

$$
\begin{aligned}
a_\infty(V) &= \frac{1}{1+exp(-(V+14)/16.6)} \\
\tau_a(V) &= 5 \\
\alpha_h(V) &= 0.07exp(-(V+63)/20) \\
b_\infty(V) &= \frac{1}{1+exp((V+71)/7.3)} \\
\tau_b(V) &= \frac{1}{\frac{0.000009}{exp((V-26)/18.5)} + \frac{0.014}{0.2+exp(-(V+70)/11)}} \\
r_\infty(V) &= \frac{1}{1+exp((V+48)/10.2)} \\
\tau_r(V) &= \frac{1}{exp(-14.59-0.086V)+exp(-1.87+0.0701V)}
\end{aligned} \tag{17}
$$

The parameter values for the above equations are $C = 1.3, g_L = 0.05, g_{Na} = 30, g_K = 23, g_A = 16, g_h = 12, V_{Na} = 90, V_K = -100, V_A = -90, V_h = -32.9, V_L = -70$.

Next, we move on to modeling the synapses in this network.

## 2 SYNAPTIC MODEL

The utilized synaptic model in this work is adopted from Ermentrout and Kopell (1998). We represent the synaptic gating variable for each synapse associated with the presynaptic neuron with $s$ where $0 \leq s \leq 1$. This variable obeys:

$$\frac{ds}{dt} = \rho(V)\frac{1-s}{\tau_R} - \frac{s}{\tau_D} \tag{18}$$

Here, $\tau_R$ and $\tau_D$ are the rise and decay time constants, respectively and $\rho$ denotes a smoothed Heaviside function as the following:

$$\rho(V) = \frac{1+tanh(\frac{V}{4})}{2} \tag{19}$$

The synaptic input to every given neuron $j$ from a neuron $i$ in the model must be added to the right-hand side of the equation which governs the membrane potential $V_j$. This term is added in the form of:

$$g_{ij}s_i(t)(V_{srev} - V_j) \tag{20}$$

where $g_{ij}$ denotes the maximal conductance associated with the synapse, $s_i$ represents the gating variable associated with neuron $i$, and $V_{srev}$ is the synaptic reversal potential. For $AMPA$ receptor-mediated synapses, we have $\tau_R$ = 0.1, $\tau_D$ = 3, and $V_{srev}$ = 0; for $GABA_A$ receptor-mediated synapses, $\tau_R$ = 0.2, $\tau_D$ = 20, and $V_{srev}$ = -80, if the synapse originates from an O-cell.

## 3 PARAMETER SET

Here we bring the parameter values utilized in this work to model the hippocampal network which behaves as a PING network with nested gamma and theta rhythms. The model LFP exhibits mixed gamma and theta oscillations as observed experimentally in coronal slices of hippocampus Gloveli et al. (2005).
Let us represent the number of E, I, and O-cells with $N_E$, $N_I$, and $N_O$. We denote the maximal conductance of the synaptic connection between cell type $X$ and cell type $Y$ with $G_{XY}$.

$$G_{XY} = \frac{g_{XY}}{N_X}, \qquad X, Y = O, I, E. \tag{21}$$

the employed $g_{XY}$ for each synapse type between different cell types are: $g_{II} = 0.1$, $g_{OI} = 0.15$, $g_{IO} = 0.5$, $g_{IE} = 0.08$, $g_{OE} = 0.15$, $g_{EI} = 0.05$, and $g_{EO} = 0.01$ in $\frac{mS}{cm^2}$. All unspecified parameters are equal to zero.

## 4 ADDITIONAL RESULTS

In this section, we further explored the fluctuation of the network oscillations in response to variations in the drive currents $I_E$ and $I_I$. We investigate the current value range between 0.2 and 1 $\frac{\mu A}{cm^2}$ for both $I_E$ and $I_I$. In this section, we only include the simultaneous effect of varying these currents concurrently with varying $g_A$, $g_H$, and $g_{KDRe}$ conductances as sample case studies. Due to the increased complexity with the addition of drive current variations, further analysis of these results is out of the scope of this study.
Figures 1 and 2, represent the results comparing network oscillations in theta and gamma ranges while varying drive currents to E-cells and I-cells as well as $g_A$, $g_H$, and $g_{KDRe}$. The theta range oscillations show higher vulnerability to drive current changes compared to gamma oscillations. This difference is more significant in the case of varying drive currents concurrently with $g_{KDRe}$ where the theta peak is nearly lost at many variations of $I_E$ and $I_I$ (Figures 1f and 2f). However, the power of the peak at gamma range is much less impacted by the changes in drive currents and this peak seems to be more robust in the network in all study cases of $g_A$, $g_H$, and $g_{KDRe}$. These figures also confirm the expectations from a PING network discussed in the Introduction section where very weak $I_E$ or strong $I_I$ values abolish the PING rhythms and disturb the network activity.
Due to the extra complexity by the addition of drive current variations, we leave further discussions about the clear impact of this variable in our model to future works.

## REFERENCES

Ermentrout, G. B. and Kopell, N. (1998). Fine structure of neural spiking and synchronization in the presence of conduction delays. *Proceedings of the National Academy of Sciences* 95, 1259–1264

Gloveli, T., Dugladze, T., Rotstein, H. G., Traub, R. D., Monyer, H., Heinemann, U., et al. (2005). Orthogonal arrangement of rhythm-generating microcircuits in the hippocampus. *Proceedings of the National Academy of Sciences* 102, 13295–13300

Li, G. and Cleland, T. A. (2013). A two-layer biophysical model of cholinergic neuromodulation in olfactory bulb. *Journal of Neuroscience* 33, 3037–3058

Olufsen, M. S., Whittington, M. A., Camperi, M., and Kopell, N. (2003). New roles for the gamma rhythm: population tuning and preprocessing for the beta rhythm. *Journal of computational neuroscience* 14, 33–54

Saraga, F., Wu, C., Zhang, L., and Skinner, F. (2003). Active dendrites and spike propagation in multicompartment models of oriens-lacunosum/moleculare hippocampal interneurons. *The Journal of physiology* 552, 673–689

Tort, A. B., Rotstein, H. G., Dugladze, T., Gloveli, T., and Kopell, N. J. (2007). On the formation of gamma-coherent cell assemblies by oriens lacunosum-moleculare interneurons in the hippocampus. *Proceedings of the National Academy of Sciences* 104, 13490–13495

Wang, X.-J. and Buzsáki, G. (1996). Gamma oscillation by synaptic inhibition in a hippocampal interneuronal network model. *Journal of neuroscience* 16, 6402–6413

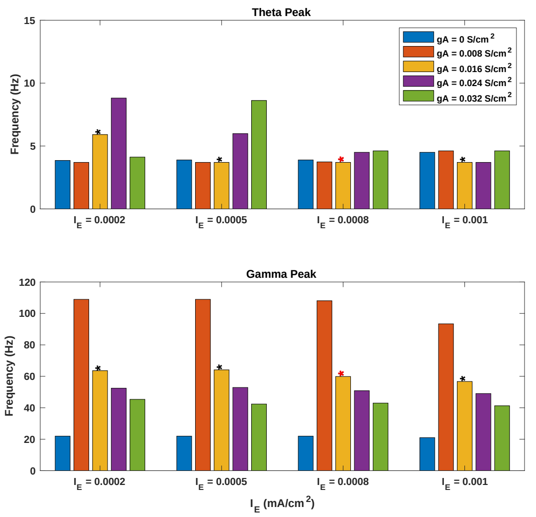


**a) Frequency changes with $I_E$ and $g_A$ variations**

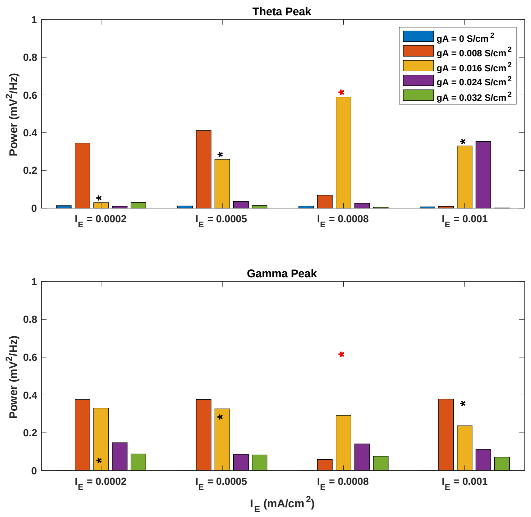


**b) Power changes with $I_E$ and $g_A$ variations**

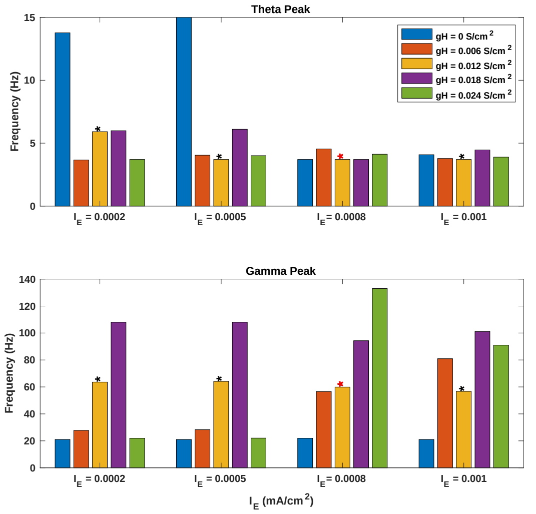


**c) Frequency changes with $I_E$ and $g_H$ variations**

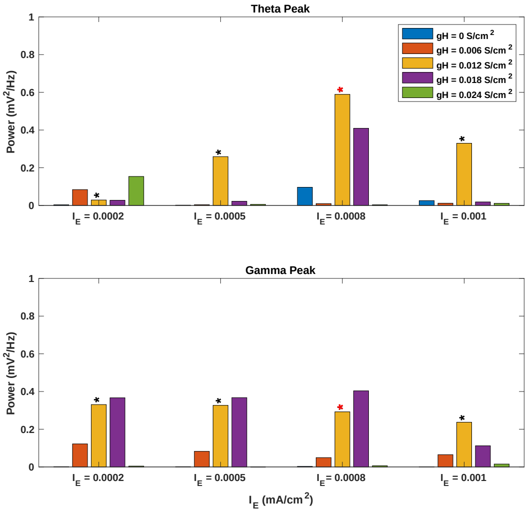


**d) Power changes with $I_E$ and $g_H$ variations**

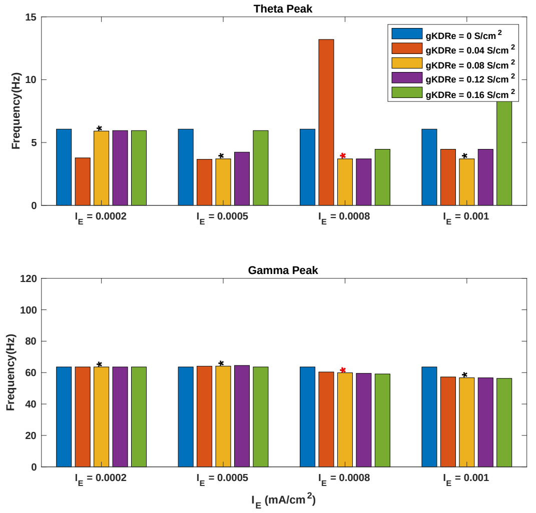


**e) Frequency changes with $I_E$ and $g_{KDRe}$ variations**

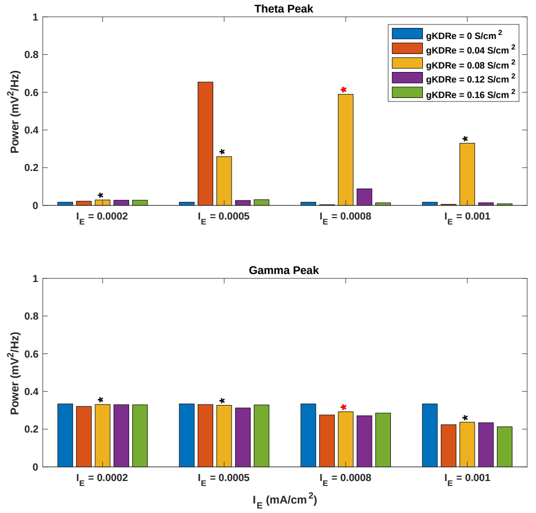


**f) Power changes with $I_E$ and $g_{KDRe}$ variations**

**Figure 1. Effects of changing $I_E$ and conductances simultaneously.** The black asterisks represent baseline conductance values at each $I_E$ level and the red asterisks indicate all variables (conductances and drive currents) being at baseline. Graphs also display three adjusted drive currents for each conductance level for **(a,b)** $g_A$, **(c,d)** $g_H$, and **(e,f)** $g_{KDRe}$. Fluctuations in peak frequency (left) and power (right) are given at each conductance and current pair for theta (top) and gamma (bottom) rhythms.

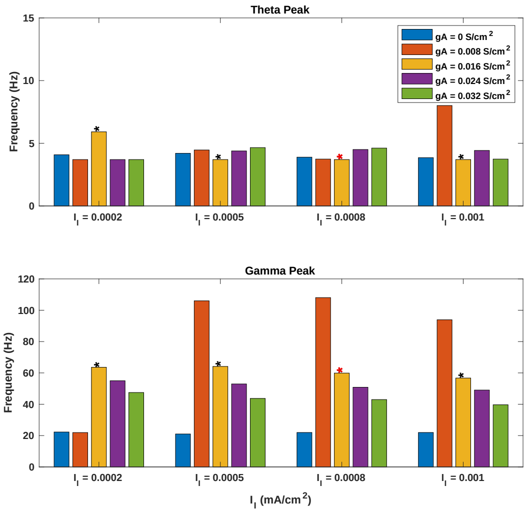


**a) Frequency changes with $I_I$ and $g_A$ variations**

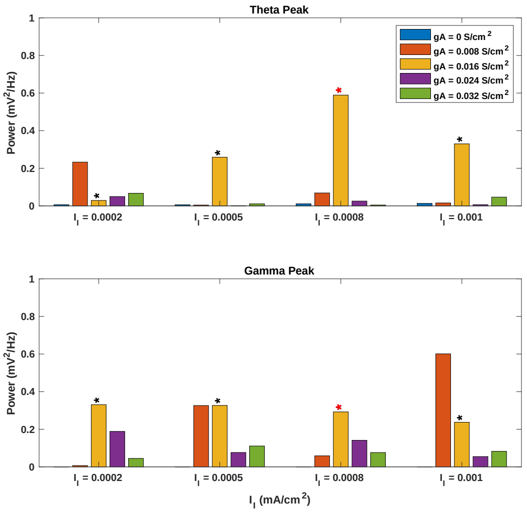


**b) Power changes with $I_I$ and $g_A$ variations**

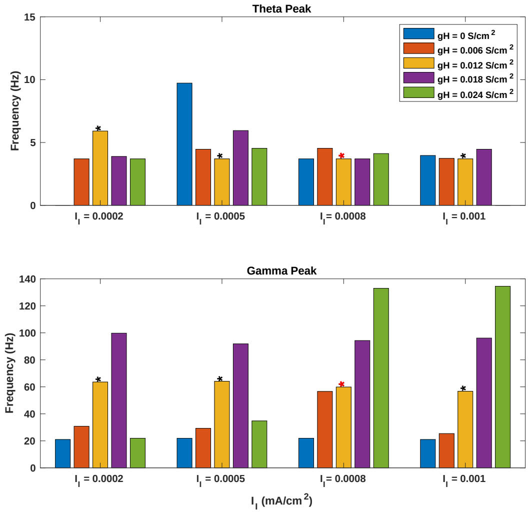


**c) Frequency changes with $I_I$ and $g_H$ variations**

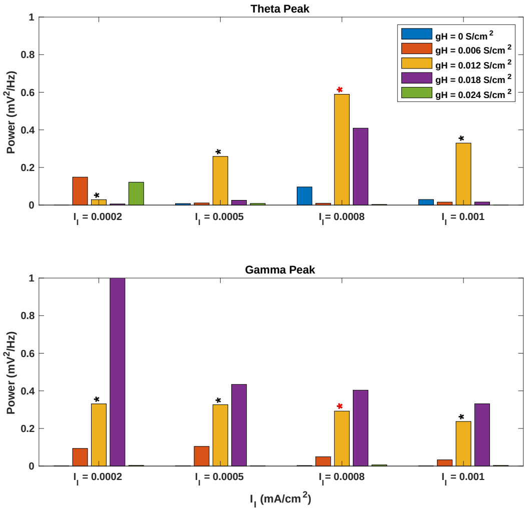


**d) Power changes with $I_I$ and $g_H$ variations**

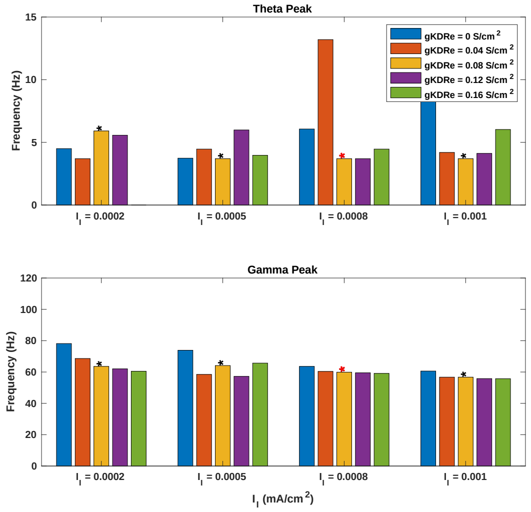


**e) Frequency changes with $I_I$ and $g_{KDRe}$ variations**

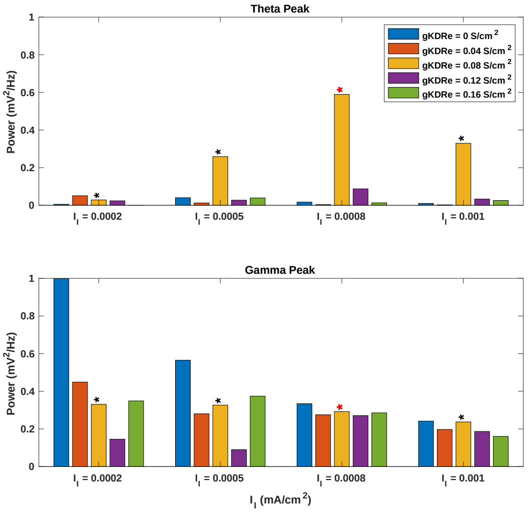


**f) Power changes with $I_I$ and $g_{KDRe}$ variations**

**Figure 2. Effects of changing $I_I$ and conductances simultaneously.** The black asterisks represent baseline conductance values at each $I_I$ level and the red asterisks indicate both conductances and drive currents being at baseline. Graphs also display three adjusted drive currents for each conductance level for **(a,b)** $g_A$, **(c,d)** $g_H$, and **(e,f)** $g_{KDRe}$. Fluctuations in peak frequency (left) and power (right) are given at each conductance and current pair for theta (top) and gamma (bottom) rhythms.
